# Gene‐to‐Population Level Responses to Multiple Stressors on the Rocky Shore

**DOI:** 10.1002/ece3.73368

**Published:** 2026-04-07

**Authors:** Ramesh Wilson, Katie Driver, James Orr, Manuela Truebano, Michael Collins, Michelle Jackson

**Affiliations:** ^1^ Department of Biology University of Oxford Oxford UK; ^2^ School of the Environment The University of Queensland Brisbane Australia; ^3^ School of Biological and Marine Sciences University of Plymouth Plymouth UK

**Keywords:** coastal ecology, functional ecology, global change, multiple stressors, nutrient enrichment, warming

## Abstract

Coastal ecosystems are exposed to both global and local stressors operating across multiple scales. However, research rarely considers how their combined effects propagate over time and across levels of biological organisation. Here, we employ an in situ warming experiment across two rocky shore sites with contrasting sewage pollution to quantify independent and interactive effects of warming and nutrient pollution from genes to populations. Passively warmed and control settlement plates were deployed at polluted and non‐polluted sites and surveyed across a summer to quantify temporal dynamics in the responses of key intertidal taxa. Barnacles were further employed as a model for comparing responses across biological levels, including body size, stable isotope analysis and RNA sequencing. Pollution consistently increased invertebrate abundance and macroalgal cover, alongside transient positive effects on barnacle size, whereas warming reduced barnacle abundance and suppressed macroalgal cover late in the season. Warming and pollution interacted synergistically on barnacle abundance, with pollution remaining the dominant stressor. Microphytobenthos groups similarly showed distinct pollution‐driven increases with warming primarily modifying temporal trajectories; cyanobacteria showed both date‐specific and season‐wide synergistic interactions, against a backdrop of temporal variability across stressor treatments. Consistent with these patterns, pollution shifted barnacle δ^13^C and δ^15^N towards values indicative of greater assimilation of sewage‐derived material, while warming increased elemental C:N ratios, consistent with altered nutritional stress. Barnacle transcriptomic responses mirrored this dominance of pollution, broadly regulating gene expression linked to protein turnover, DNA repair and protein folding; combined warming and pollution further intensified proteostasis‐related changes and produced predominantly reversal and antagonistic interaction types. Our results show that sewage pollution can overwhelm and reshape warming effects over time and across biological levels, linking group‐level responses with parallel shifts in trophic biomarkers and gene regulation. Our scalable field approach provides a template for in situ marine multiple stressor experiments across wider spatiotemporal scales.

## Graphical abstract

This experiment employs an in situ field experiment on a rocky shore to test how simulated warming and ambient sewage‐associated nutrient pollution independently and interactively affect ecological responses from gene expression to functional‐group dynamics across a summer season. The polluted‐site contrast was the dominant driver of ecological and molecular responses, increasing barnacle abundance and several algal metrics, while warming generally reduced barnacle abundance and suppressed macroalgal cover late in the season. Interaction effects were common but time‐ and organisational level‐dependent, showing that local pollution context can reshape warming responses across biological scales.

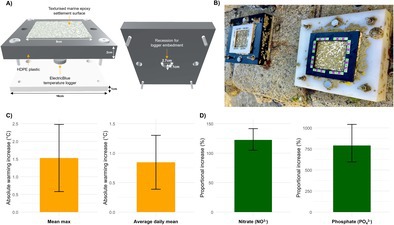

## 1 Introduction

Anthropogenic stressors are rapidly degrading natural ecosystems. These stressors include both global drivers, such as climate change‐related processes including warming and ocean acidification, and local pressures, such as nutrient pollution, tourism and land use. As a result, multiple stressor research has rapidly emerged as a key area of concern in global change biology (Orr et al. 2020), aiming to understand the combined and potentially interactive effects of co‐occurring stressors on ecosystem structure and function. Combined stressor effects may be additive, or may deviate from additivity through synergistic or antagonistic interactions, whereby the net response is greater or smaller than expected from the independent effects of each stressor alone. Such non‐linear impacts are a major concern for management strategies due to their often surprising and potentially detrimental outcomes. Greater monitoring and research efforts are necessary to mitigate such ‘ecological surprises’ (Kunze et al. 2021; Pansch and Hiebenthal 2019), through adequate identification and management of their underlying mechanisms.

Marine multiple stressor studies have increased over the last decade, focusing largely on cumulative climate impacts, generalised pollution and fisheries (Bass et al. 2021). However, there remains a significant gap in upscaling research from single‐species responses to population‐, functional‐group‐ and ecosystem‐level responses, particularly where stressors operate across different spatial scales (Cabral et al. 2019; Crain et al. 2008; Hoppit and Schmidt 2022). While almost 75% of studies have shown that climate change intensifies the impacts of local stressors (Gissi et al. 2021), particularly at the species level, these findings lack consistent generalisability across higher levels of biological organisation and environmental context (Collins et al. 2023; Gissi et al. 2021; Turschwell et al. 2022). This highlights the need to consider local context dependence and ecosystem scale when interpreting research findings into practical management.

Rocky shores are particularly well suited to addressing these questions because they are tractable, experimentally accessible systems in which strong environmental variability, intense space competition and relatively rapid turnover can reveal stressor effects across ecological levels (Hawkins et al. 2020, 2025; Kunze et al. 2021; Sanz‐Lazaro et al. 2022). Intertidal organisms experience both marine immersion and aerial exposure, making them useful for understanding how biological responses emerge under strongly fluctuating thermal, desiccation and hydrodynamic regimes across tidal zones (Helmuth et al. 2006; Kordas et al. 2015; Pansch and Hiebenthal 2019). Moreover, understanding stressor‐response dynamics in intertidal ecosystems is relevant beyond rocky shores as the land‐sea interface links land‐based nutrient and contaminant delivery, habitat connectivity and ecosystem services that support fisheries, water quality, recreation and shoreline protection (Fang et al. 2018; Singh et al. 2021).

Intertidal systems experience strong diurnal and seasonal variation in environmental parameters (Kunze et al. 2021). Their zonation reflects gradients in physiological tolerance and biotic interactions, while persistence under variable conditions is further shaped by physiological plasticity, ecological memory, generation time and dispersal (Mieszkowska et al. 2019). However, despite these adaptive capacities, this innately stressful and variable environment may compound the impacts of additional anthropogenic stressors. Climate‐related drivers often operate across regional to global scales and are driven by processes beyond local management control. As a result, coastal management often focuses on more tangible local stressors such as water quality and land encroachment in isolation (Kunze et al. 2021; Wedding et al. 2022).

Combining local and global stressors is therefore especially important in coastal environments, where land‐based pollution can modify how climatic stress is experienced and expressed. Treated and untreated sewage discharges from combined sewer overflows can increase nutrient and organic matter loading, contribute to eutrophication and oxygen depletion and alter coastal trophic dynamics (Cabral‐Oliveira et al. 2014; Giakoumis and Voulvoulis 2023; Perry et al. 2024). In England, concern over such inputs has intensified in recent years owing to frequent storm‐overflow releases and ageing or under‐capacity wastewater infrastructure (Giakoumis and Voulvoulis 2023; Perry et al. 2024). These local stressors, against a backdrop of global drivers like climate change, create complex interactions that require further research to understand their combined effects. There is a clear need to quantify multiple stressor impacts in a format that is applicable to policy and management, with a particular focus on coastal environments.

Stressor effects can differ markedly across biological levels, from gene expression and physiology to populations and ecological groups, even within the same system. Linking multiple levels of biological organisation is critical for understanding how stressor effects emerge and propagate (Kong et al. 2025; Simmons et al. 2021). At the organismal level, stress responses can alter maintenance, growth, feeding and reproduction; these changes can then influence abundance, trophic interactions and the relative dominance of ecological groups over time (Ames et al. 2020; Jackson et al. 2021). Yet, the vast majority of multiple stressor studies still focus on single response levels, limiting inference about how local and global drivers jointly influence organismal performance and higher‐level ecological structure (Gissi et al. 2021; Orr et al. 2024; Turschwell et al. 2022).

The aim of this study was therefore to quantify the impacts of multiple stressors across levels of biological organisation in a rocky shore ecosystem, from gene expression and trophic biomarkers to population‐ and functional‐group‐level responses. To achieve this, an in situ warming experiment was installed across two rocky shore sites contrasting in ambient sewage‐associated nutrient pollution. We evaluated the independent and interactive effects of warming and pollution on key biotic functional groups (barnacles, grazing gastropods, macroalgae, microphytobenthos) throughout a summer season. Barnacles were additionally employed as a model group to investigate population responses through body size, trophic ecology assessed using stable isotope analysis and transcriptional state assessed using RNA‐seq. Given their sessile nature and roles as abundant suspension feeders, dominant space occupiers and habitat‐modifying intertidal organisms, barnacles serve as an ideal system for assessing whether stressor effects align or decouple across biological levels. The following a priori predictions were tested:

### 1.1 Predicted Effects of Warming

Warming is expected to negatively affect all sessile groups due to increased energetic demands and physiological strain (Bernatchez et al. 2019; Kordas et al. 2015), whereas effects on motile grazers occupying the plates may be weaker or more transient if movement reduces sustained exposure to warmed surfaces. Reduced grazing pressure may further benefit macroalgae through reduced herbivory (Kordas et al. 2015). Stable isotope signatures of barnacles are not expected to respond strongly to warming, given that the treatment is localised to the substrate and does not affect the water column and its food sources, although warming could still influence feeding behaviour or particle capture efficiency (Maar et al. 2024). We therefore expected any isotopic shifts associated with warming to be weaker than those driven by pollution‐linked changes in food source or availability. However, warming may impose physiological stress on barnacles, reflected by an increased elemental C:N mass ratio (i.e., the mass ratio of elemental carbon to nitrogen in tissues), indicating shifts in tissue composition under thermal stress (Zhang et al. 2018). At the transcriptional level, we expect warming to induce activation of cellular stress pathways and metabolic reallocation. Given the temperature contrast (see Methods) and exposure prior to sampling, any initial acute stress response is likely to be muted, with RNA‐seq primarily capturing the longer‐term, chronic transcriptional state (Collins et al. 2023; López‐Maury et al. 2008; Zhan et al. 2019).

### 1.2 Predicted Effects of Pollution

Under the polluted site conditions, macroalgal cover is predicted to correlate positively with greater nutrient availability, in line with typical eutrophication trends, while simultaneously providing abundant food sources for grazing gastropods (Alestra and Schiel 2015; Kordas et al. 2015). Polluted conditions may also trigger phytoplankton blooms, increasing food availability for sessile barnacles, which do not rely on local benthic primary productivity at the plate level (D'Costa et al. 2017). Elevated nutrients are expected to shift barnacle trophic dynamics, reflected by changes in stable isotope ratios that indicate alterations in the composition and source of available food (Laitano et al. 2018). At the transcriptional level, we expect nutrient enrichment (together with co‐discharged contaminants associated with sewage inputs) to induce a broad response dominated by the upregulation of xenobiotic‐ and stress‐response pathways, as well as metabolic processes reflecting higher energy intake (Balbi et al. 2021; Schaefer et al. 2022).

### 1.3 Predicted Interactive Effects of Warming and Pollution

We expect a net negative impact on macroalgae due to thermal stress, partly mitigated by pollution, as documented in previous studies (Borburema et al. 2020; Charan et al. 2022). Similarly, sessile barnacles may be negatively impacted by warming and its associated rise in metabolic demand, but this may be offset by increased food availability in polluted conditions. Functional groups may also benefit from removal of other groups, alleviating spatial competition (Crain et al. 2008; Harvey et al. 2013). Grazing gastropods may exploit abundant food (Kordas et al. 2015), but any interaction with warming may be dampened if grazers are not persistently exposed to the warmed plate surfaces due to their motility. Barnacle trophic indicators are expected to primarily reflect pollution‐induced shifts in nutrient and food‐web dynamics, with no additional impact from warming. At the transcriptional level, we predict that combined warming and pollution will alter the magnitude and nature of pollution‐induced responses, with upregulation of genes related to energy metabolism and ATP production and a relative downregulation of protein‐folding pathways compared to warming alone, reflecting interaction effects on cellular energetics and proteostasis (i.e., maintenance of protein homeostasis) (Brasseur et al. 2022; Collins et al. 2023; Mohamed et al. 2014).

## 2 Materials and Methods

### 2.1 Experimental Design

We conducted our experiment in Peacehaven in East Sussex, England. Tides in this area are mixed semidiurnal, with a mean spring tidal range of ~6.3 m at nearby Newhaven (New Forest District Council 2017). The region is classified as Cfb (temperate oceanic climate; Köppen‐Geiger classification), characterised by a maritime climate with a narrow annual temperature range. Such conditions make the region an ideal system to investigate heightened thermal stress on intertidal ecosystems. Peacehaven is surrounded by multiple sewage outfalls operated by Southern Water (Supporting Information (SI); Figure S1), with growing public concern surrounding pollution and water treatment misconduct (Cipirska 2021; Department for Environment, Food, and Rural Affairs 2023). Permission for installation was provided by Natural England and the Crown Estate.

**FIGURE 1:**
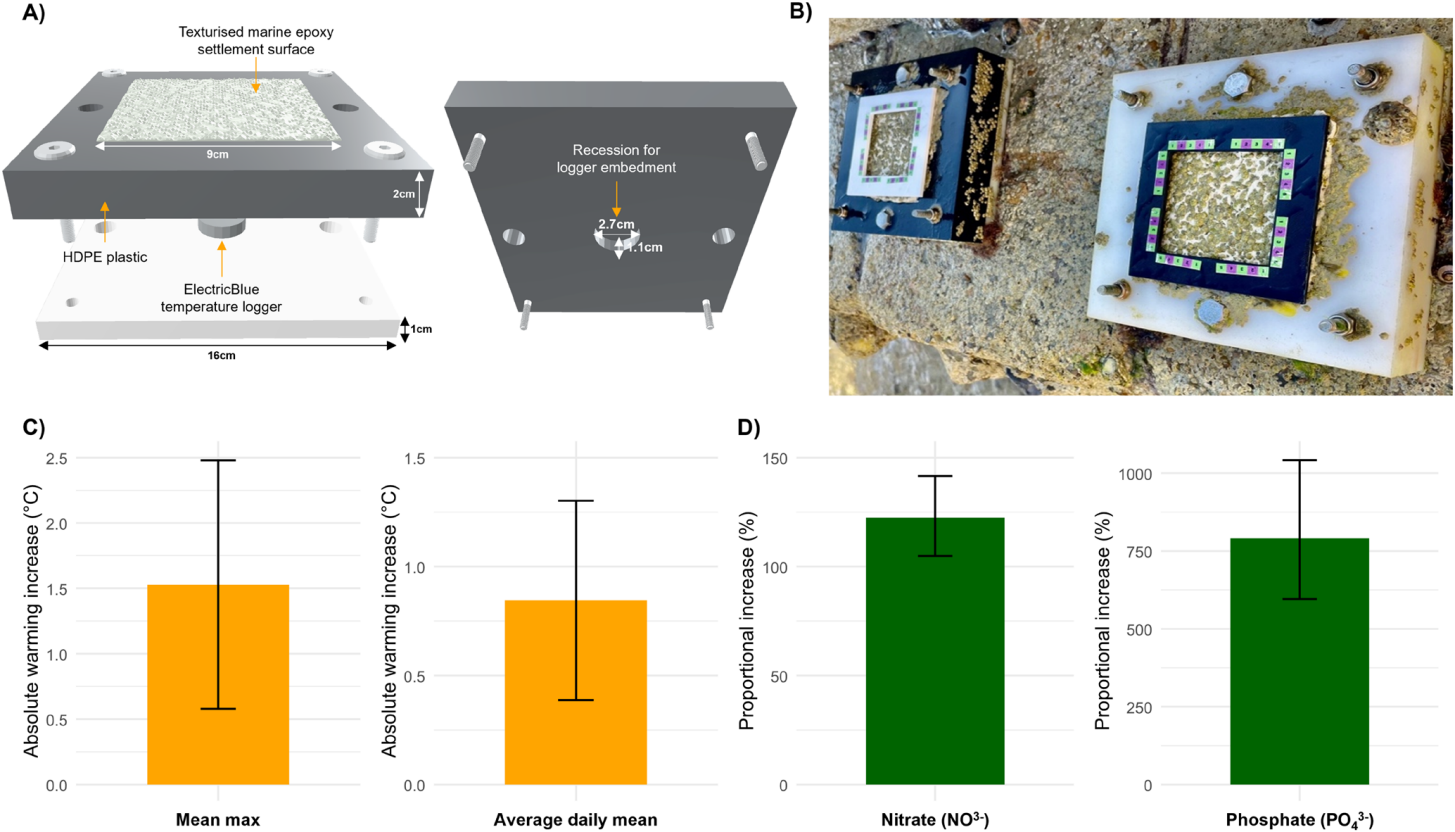
Summary figure of methodology. (A) 3D rendering of the black (warmed) treatment plate, showing the epoxy settlement surface on the left and the underside of the top plate with recession for installation of the temperature logger on the right. (B) 3D‐printed 40% opening sampling stencil, shown here on developed black and white plates, with 2.5 cm scale bars applied for barnacle size measurement calibration. (C) Absolute warming increases (°C), shown as two separate warming metrics: Mean maximum temperature and tide‐adjusted average daily mean for the warmed treatment relative to the ambient control (Δ*T* = Warmed—Ambient), recorded and averaged across all mid‐tide plates during the experimental period (*n* = 12 warmed plates; *n* = 12 ambient plates; see SI for full warming verification). Error bars show 95% confidence intervals for ΔT, based on the difference between independent treatment means, with uncertainty propagated from the two groups' standard errors. (D) Proportional increase (%) in mean verification values of nitrate and phosphate at the polluted site relative to the non‐polluted control, based on three paired pre‐installation verification excursions (*n* = 3 paired excursions; each excursion based on the mean of three water samples per site, i.e., 9 raw samples per site overall). Nitrate and phosphate are shown in separate subpanels with independent y‐axis scales, because these metrics differ markedly in magnitude; bar heights should therefore be interpreted relative to the axis within each subpanel rather than compared directly between nutrients. For nitrate, absolute concentrations are not reported because the in situ ion‐selective electrode (ISE) was affected by chloride in seawater; we therefore show proportional elevation under matched PSU (salinity) between sites (see SI). Phosphate is shown the same way for comparability, and its absolute verification values are provided in SI Table S1.

We employed a fully factorial 2 × 2 experimental design with passively heated settlement plates in two sites with contrasting nutrient pollution levels. To create our warming condition, we constructed 12 control (white) and 12 warmed (black) plates (Figure 1A,B), inspired by Kordas et al. (2015), with colour ensuring a categorical difference in thermal absorption when installed at mid‐tide (Figure 1C), as shown in similar studies (Kordas et al. 2015; Kordas and Harley 2016; Lathlean and Minchinton 2012). A full instructional document for plate construction is provided in the SI. Six black and six white plates were deployed in each site (polluted vs. non‐polluted). The polluted site had significantly elevated levels of both dissolved inorganic nitrate (NO^3−^) and dissolved inorganic phosphate (PO_4_^3−^) (Figure 1D; see SI Figure S2 for verifications during the experimental period). Because only one site represented each pollution context, pollution is interpreted here as a site‐level contrast in ambient sewage‐associated nutrient conditions rather than as a spatially replicated pollution treatment. Full information on temperature monitoring, settlement plate design and nutrient quantification prior to and during the experimental period is provided in the SI (Supplementary Methods (Experimental); Tables S1 and S2; Figure S2).

Each plate was topped with a 9 cm × 9 cm textured epoxy settlement area, mimicking natural rocky substrate for recruitment (Kordas et al. 2015). All plates were installed at mid‐tide (established via typical biotic zonation cues and local tidal height markers), standardising aerial exposure which drives the daily warming difference. Plates were mounted horizontally, with the settlement surface facing upward, on near‐horizontal substrata at both sites. To record settlement temperature, ElectricBlue EnvLoggers (T2.4 hardware, 27 mm format) were embedded within the plates in direct contact with the epoxy, recording temperature every 90 min (0.1°C resolution) (Figure 1A). Plates were installed in January 2023 and left in place until June 2023 to permit field establishment of naturally recruiting assemblages under continuous treatment exposure before repeated summer surveys, after which seven fortnightly surveys were conducted from June–September 2023, following similar in situ schedules (Kordas et al. 2015; Kordas and Harley 2016).

### 2.2 Sampling

At each of the seven sampling dates, we quantified four functional‐group responses on settlement areas: (1) barnacle abundance; (2) grazing gastropod abundance; (3) macroalgal cover; and (4) microphytobenthos concentration (cyanobacteria, diatoms, green microalgae). Data were obtained via in situ counts/assessments and photo‐based analyses, allowing sampling under time‐limited tidal windows.

#### 2.2.1 Season‐Wide Functional‐Group/Population Responses

For barnacle abundance, populations were subsampled using a stencil with a 40% opening (Figure 1B), to discount edge effects. Photo images were then analysed using a machine‐learning model produced by the mobile application ‘CountThings’, which employs convolutional neural networks (CNNs) alongside specialised one‐stage, two‐stage, keypoint‐based and transformer‐based frameworks for robust object detection. The model was trained on marked field‐establishment images and distinguished live barnacles via intact opercula (see Supplementary Methods (Experimental) for further details on CountThings architecture and settings). Sampling stencils contained a 25 mm scale bar used to calibrate barnacle body size measurements in ImageJ (Figure 1B). Images were overlaid with a 10 × 10 grid, and 10 random coordinates were drawn; at each coordinate, the upper‐leftmost barnacle was measured across the maximum rostro‐carinal axis following previous methods employed as a non‐destructive, allometric proxy (Ten et al. 2019). New images were taken for macroalgae coverage analyses without a stencil. Colour thresholding metrics were applied in ImageJ (RGB: Red: 0–50; Green: 0–250; Blue: 0–50), maximising green pigments whilst minimising hues from invertebrates and epoxy.

For microphytobenthos, a BenthoTorch (bbe Moldaenke) was used on each plate. The BenthoTorch permits rapid quantification of diatoms, green microalgae and cyanobacteria through excitation of each group's characteristic fluorescence profile (chl‐a/cm^2^). Plates were shaded for 30 min before measurement to stabilise light‐driven variation in chlorophyll‐a (Kaylor et al. 2018).

#### 2.2.2 End‐of‐Summer Responses: Barnacles

At the end of summer, two barnacles of the same species ( *Semibalanus balanoides* ) per settlement plate were randomly selected for stable isotope analysis (SIA). Internal tissues were scraped into 1.5 mL microcentrifuge tubes (48 samples), dried at 60°C for 48 h, and weighed. Tissues were then pooled by plate into 24 composite samples (*n* = 6 per treatment; two samples below the recommended ≥ 0.7 mg threshold for laboratory analyses). Samples were analysed using a SerCon Callisto CF‐IRMS system to obtain δ^13^C (Vienna Pee Dee Belemnite standard) and δ^15^N (atmospheric N₂ standard) values, from which elemental C:N ratios were derived. Intermittent runs of calibration standards (seal collagen, alanine and IAEA‐CH‐6 (sucrose)) provided continuous drift correction and verified instrument performance, with seal collagen and alanine chosen for their stable, well‐characterised isotopic compositions and IAEA‐CH‐6 serving as an additional δ^13^C standard.

For barnacle gene expression analyses, two barnacles were similarly selected per plate at the end of summer for RNA sequencing (RNA‐seq), and preserved in RNAlater solution (Invitrogen) according to the manufacturer's instructions. This end‐of‐summer sampling was intended to characterise the integrated late‐season transcriptional state after repeated treatment exposure, rather than an acute response to a single emersion event (see Supplementary Methods (Experimental)). Samples stored in RNAlater were rinsed in phosphate‐buffered saline (PBS) to remove precipitated salts, and RNA was then isolated from individual barnacles using a RNeasy Micro Kit (Qiagen, Hilden, Germany), according to manufacturer instructions. RNA quantity, purity and integrity were assessed using an Agilent 5400 Bioanalyzer. Samples with an RNA Integrity Number (RIN) > 5, indicating suitably low degradation for laboratory analyses (*n* = 8 for ambient × polluted/non‐polluted; *n* = 7 for warmed × polluted/non‐polluted), were subjected to RNA‐seq on one lane of an Illumina NovaSeq X Series (paired‐end (PE) 150 bp) by Novogene, Cambridge, UK. Assembly was performed by Novogene, with subsequent bioinformatics analyses performed in‐house.

### 2.3 Statistical Analyses

Statistical analyses of ecological data were run in R version 4.4.3.

#### 2.3.1 Season‐Wide Functional‐Group/Population Responses

To evaluate the independent and interactive effects of warming and nutrient pollution, we used two modelling approaches. For each sampling date, generalised linear models (GLMs) with appropriate families and links were fitted for each response (model specifications and diagnostics summarised in SI Table S3). Additionally, to capture season‐long trends in stressor interactions and responses, generalised additive mixed models (GAMMs) were constructed for every response variable. Each GAMM incorporated smooth terms for time (days since the start of summer) and allowed interactions with each of the categorical stressor variables, while also including plate identity as a random effect to account for repeated measures (see Supplementary Methods (Analyses)). GAMMs are widely used to model non‐linear temporal trends in ecological time series, including irregularly spaced observations and seasonal trajectories (Pedersen et al. 2019; Simpson 2018; Wood 2025). Diagnostics confirmed residual normality and homoscedasticity. Notably, for grazers, data were aggregated to binary presence/absence (i.e., occurrence) to improve GLM and GAMM model convergence, given the high frequency of zero counts and separation issues; resulting log‐odds models are thus interpreted as the probability of grazer presence in a given treatment (see Supplementary Methods (Analyses)). Furthermore, conditional plots were constructed for all GLMs to visualise effect modification and asymmetry in interactions, showing directly whether the effect of warming differed between pollution treatment sites on each sampling date, as recommended for detecting and interpreting interactive ecological responses (Spake et al. 2023). Full details on GLM/GAMM model choices, diagnostics and conditional plots are provided in the SI (Supplementary Methods (Analyses); Table S3; Figures S3–S9).

To classify interaction effects, we extracted the relevant coefficient estimates from our GLM and GAMM models, and interpreted the fitted regression interaction as a departure from a null model of independent stressor effects on the model's link scale (Madge Pimentel et al. 2025). Each model's linear predictor comprises the intercept (*α*), the main effect coefficients for warming (*β*(w)) and pollution (*β*(p)) and an interaction term (*β*(int)). We define the ‘additive’ expectation (i.e., the null model on a given model's link scale) as the sum of the main effect coefficients (Δadd = *β*(w) + *β*(p)). We then define the observed change including the interaction (Δobs = Δadd + *β*(int)), where Δobs represents the total predicted change when the interaction between warming and pollution is accounted for. An interaction coefficient of zero indicates that the observed response matches this null expectation, whereas any deviation (*β*(int) ≠ 0) quantifies how much the combined effect diverges from this. Notably, because ‘additivity’ is scale‐dependent, interaction classifications are specific to the link function used (Madge Pimentel et al. 2025; Spake et al. 2023). Moreover, we note that such interactions are statistical (effect modification in the fitted model), and do not definitively evidence unique mechanistic interactions between stressors (Orr et al. 2024; Schäfer et al. 2023; Spake et al. 2023); we therefore interpret interaction types cautiously and in the context of biological plausibility and experimental design. For interpretive clarity, the interaction term therefore indicates whether the effect of warming was stronger, weaker, or directionally altered at the polluted site relative to the null expectation from the main effects alone.

The interpretation of the statistical interaction term is contextualised by the direction of the independent effects. If both stressor effects are positive, a positive interaction term indicates that the observed effect exceeds the additive expectation (synergism), while a negative interaction indicates antagonism. Similarly, if both stressors are negative, a negative interaction term signifies that the combined negative impact is more severe than predicted (synergism in the negative direction), whereas a positive interaction term indicates antagonism. For stressors with opposing effects, if the interaction term shares the same direction sign as the additive expectation, the interaction reinforces the net effect (synergism relative to additivity). Whereas, if the interaction is of the opposite sign, it counteracts the net effect (antagonism). We further include the concept of a reversal (Jackson et al. 2016). A reversal occurs when the interaction term flips the overall direction of the response relative to the null expectation. For example, if both independent stressors are negative (predicting a net decrease) but the inclusion of a positive interaction term results in an overall positive response, this constitutes a reversal, and vice versa in the opposite conditions.

We further contextualised interactions using a confidence interval‐based, smallest‐effect size of interest (SESOI) comparison, grounded in equivalence‐testing logic (Lakens 2017). For models with log or logit links, additivity is defined on the link scale; after back‐transformation this corresponds to exp.(*β*(int)) = 1, where exp.(*β*(int)) is an interaction ratio that quantifies the multiplicative deviation of the combined effect from the additive expectation on the link scale (for log links, a multiplicative deviation on the response scale; for logit links, a ratio of odds ratios). We employed an SESOI band of ±5% around the null (±5% on the raw identity scale; 0.95–1.05 on the exp.(*β*(int)) scale for log and logit models) as a qualitative flag for relatively mild departures from additivity. Accordingly, when an interaction was statistically supported (*p* < 0.05), we retained its directional interaction classification, but explicitly acknowledge if the 95% CI of the interaction term overlapped this ±5% band, noting it as a mild deviation from the null model.

#### 2.3.2 End‐Of‐Summer Responses: Barnacles

Barnacle stable isotope responses were analysed once at the end of summer. Here, we modelled δ^13^C, δ^15^N, and elemental C:N mass ratio, using two‐way factorial ANOVAs with fixed effects of pollution (non‐polluted vs. polluted), warming (ambient vs. warmed) and their interaction. We report full *F*‐statistics, degrees of freedom and *p*‐values for both main and interaction terms.

For barnacle transcriptomic responses, sequencing produced ~998 million ‘clean’ reads (i.e., adaptors and low‐quality reads removed, leaving 19.5–56.2 million reads per sample), assembled into 376,108 contigs (145,062 putative genes) using Trinity v2.15.1 (Haas et al. 2013). Transcripts were annotated using the Trinotate v4.0.2 pipeline, performing BLASTx/BLASTp searches against the Swissprot and Gene Ontology (GO) databases with default parameters. To quantify gene expression, reads were aligned to the assembled transcriptome using Salmon v1.10.0 (Patro et al. 2017) with default parameters. All downstream analyses were performed in R v4.4.2. Counts were imported into R using tximport v1.34.0 (Patro et al. 2017) and differential expression was performed using DESeq2 v1.46.0 (Soneson et al. 2016). After removal of low count genes (< 10 total counts), 122,570 genes were included. The model (formula = ~Pollution + Warming + Pollution:Warming) allowed identification of significantly differentially expressed genes (DEGs, *p*_adj_ < 0.05) in response to independent warming, independent pollution and their interaction (i.e., genes whose response to warming differed between pollution conditions). For significantly interactive genes, we characterised synergisms, antagonisms and reversals as described for the ecological data. To identify affected biological pathways, we tested DEGs for gene ontology (GO) term enrichment using goseq v1.58.0 (Young et al. 2010).

## 3 Results

### 3.1 Invertebrate Responses

#### 3.1.1 Barnacle Abundance

**FIGURE 2:**
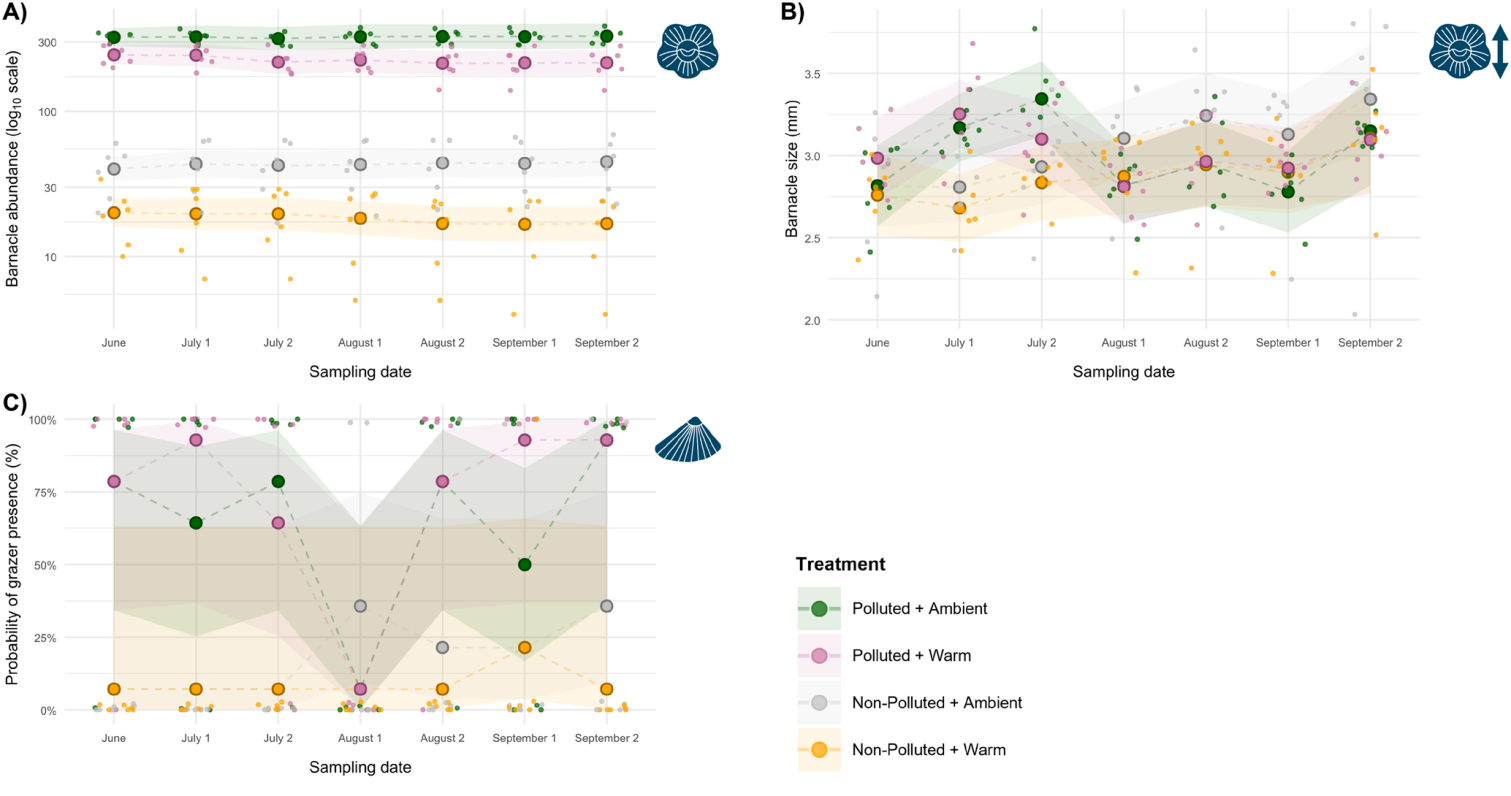
Invertebrate responses to warming across polluted and non‐polluted sites on each sampling date. Large circles show GLM model‐estimated treatment means for each sampling date; semi‐transparent ribbons show 95% confidence intervals around those date‐specific estimates, with illustrative dashed connectors between treatment means (whole‐season temporal trends are evaluated with GAMMs; see SI Figure S10 for GAMM temporal visualisations). Smaller points are jittered raw replicate values to illustrate distributions around each estimated mean response. (A) Barnacle abundance (log_10_ axis). (B) Barnacle body size (mm). (C) Grazer occurrence (predicted probability of presence). Full model specifications and diagnostics are provided in the SI (Table S3).

GLMs showed that barnacle abundance was consistently lower on warmed plates and higher at the polluted site across sampling dates. For example, in June, warming significantly reduced barnacle abundance (*β* = −0.693, SE = 0.149, *p* < 0.001), while abundance was higher at the polluted site (*β* = 2.087, SE = 0.120, *p* < 0.001). Moreover, a positive interaction term was evident (*β* = 0.414, SE = 0.182, *p* = 0.023) which, when exponentiated, corresponds to a ≈51% increase relative to the multiplicative null model (Figure 2A; SI Table S4). In direct terms, this positive interaction indicates that the negative effect of warming was weaker at the polluted site than at the non‐polluted site (equivalently, the positive site contrast was larger under warming). Notably, the positive interaction effect size on all dates remained smaller than the individual positive effect of pollution, which was the primary driver of abundance (SI Table S4). Because the additive expectation on the model (log) scale was positive on every date (Δadd = *β*(w) + *β*(p) > 0), the consistently positive interaction terms indicate amplification of that expected increase (Δobs > Δadd), and were therefore classified as synergistic deviations from the multiplicative null model on six of seven dates (+51% to +74%; SI Table S4; Figure S3). Whole‐summer GAMM predictions mirrored the discrete‐time GLM results: barnacle abundance was elevated at the polluted site (*β* = 2.087, SE = 0.210, *p* < 0.001), while warming suppressed abundance (*β* = −0.887, SE = 0.214, p < 0.001). The interaction term was positive (*β* = 0.507, SE = 0.299, *p* = 0.090), indicating near‐significant synergistic effects. However, the 95% CI ([0.924, 2.984]) overlaps our ±5% SESOI band ([0.95–1.05]); we therefore interpret the season‐wide pattern as a mild synergism. Additionally, GAMM smooth terms indicated treatment‐specific temporal structure in warmed treatments, with linear time trends (edf ~1) in the non‐polluted condition (edf = 1; *p* = 0.048), and a non‐linear temporal pattern in the polluted condition (edf = 1.802, *p* < 0.001), whereas the remaining conditions showed no detectable temporal pattern (SI Table S11; Figure S10A).

#### 3.1.2 Barnacle Size

GLMs showed no significant effect of warming on barnacle body size on any date, whereas barnacles were significantly larger at the polluted site on July 1 (*β* = 0.362, SE = 0.140, *p* = 0.017) and July 2 (*β* = 0.414, SE = 0.154, *p* = 0.014) (Figure 2B, SI Table S5). The interaction term was neither significant nor directionally consistent across dates (SI Table S5; Figure S4). The whole‐season GAMM (adjusted *R* ^2^ = 0.629) similarly showed no significance of parametric stressor terms at the season‐wide scale. However, the GAMM was dominated by significant temporal trends within treatments, with approximately linear increases in both non‐polluted treatments (Ambient: edf = 1.000, *p* < 0.001; Warmed: edf = 1.000, *p* < 0.001) and non‐linear trajectories in polluted conditions (Ambient: edf = 5.186, *p* < 0.001; Warmed: edf = 3.965, *p* < 0.001) (SI Table S12; Figure S10B).

#### 3.1.3 Grazer Occurrence

Bias‐reduced logistic GLMs indicated no independent effect of warming on grazer occurrence (log‐odds scale; interpreted as probability of presence) across dates. Grazer occurrence was, however, higher at the polluted site on two dates (June: *β* = 3.860, SE = 1.870, *p* = 0.039; the same effect was observed on July 2) (Figure 2C; SI Table S6). Interaction terms across all dates were neither consistent nor significant (SI Table S6; Figure S5). Whole‐summer GAMM predictions (adjusted *R* ^2^ = 0.602) mirrored the GLMs: occurrence was higher at the polluted site (*β* = 2.865, SE = 0.768, *p* < 0.001), warming was non‐significant (*β* = −1.640, SE = 1.225, *p* = 0.181) and the interaction was also non‐significant (*β* = 3.737, SE = 2.597, *p* = 0.150). Temporal trends varied by treatment combination, with significant smoothing terms in the ambient, non‐polluted condition (edf = 0.790, *p* = 0.050) and the warmed, non‐polluted condition (edf = 3.517, *p* = 0.024) (SI Table S13; Figure S10C).

#### 3.1.4 Barnacle Stable Isotope Composition

**FIGURE 3:**
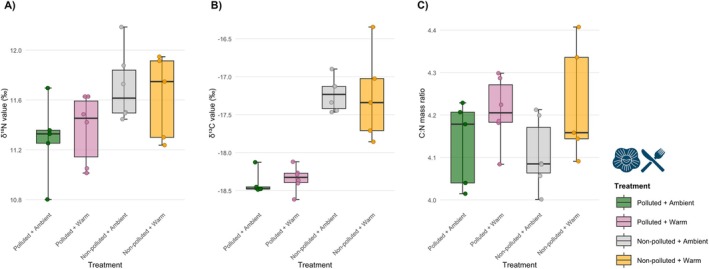
Barnacle stable isotope compositions at the end‐of‐summer sampling period (September 2). Boxes show the interquartile range (IQR), with the horizontal line indicating the median. Given the small sample size per treatment (*n* = 5–6), whiskers are shown to the minimum and maximum observed values within each treatment to aid visual interpretation, while individual points show all raw replicate values. (A) δ^15^N values (‰). (B) δ^13^C values (‰). (C) Elemental C:N mass ratio. Additional summary statistics and analytical standards outputs are provided in the SI (Tables S17 and S18).

End‐of‐summer stable isotope analyses showed site‐associated differences in δ^13^C and δ^15^N driven primarily by pollution, whereas elemental C:N mass ratio was influenced by warming (Figure 3A–C). The polluted‐site contrast was associated with lower barnacle δ^15^N, from approximately 11.67‰ in the non‐polluted site to 11.33‰ in the polluted site (*F*_1,18_ = 6.81, *p* = 0.017). Both warming and its interaction with pollution had no discernible effect on δ^15^N (*p* = 0.975 and *p* = 0.546, respectively). Similarly, barnacles at the polluted site also exhibited more negative δ^13^C, shifting from around −17.24‰ in the non‐polluted site to −18.37‰ in the polluted site (F_1,18_ = 64.90, *p* < 0.001), with no significant effect of warming or the interaction (*p* = 0.890 and *p* = 0.772, respectively). In contrast, the elemental C:N ratio was unaffected by pollution (*p* = 0.746) but increased significantly under warming, rising from ~4.12 (ambient) to ~4.22 (warmed) (F_1,18_ = 5.33, *p* = 0.033). Full group means and ANOVA outputs are provided in the SI (Tables S17 and S18).

#### 3.1.5 Barnacle Transcriptional Responses

**FIGURE 4:**
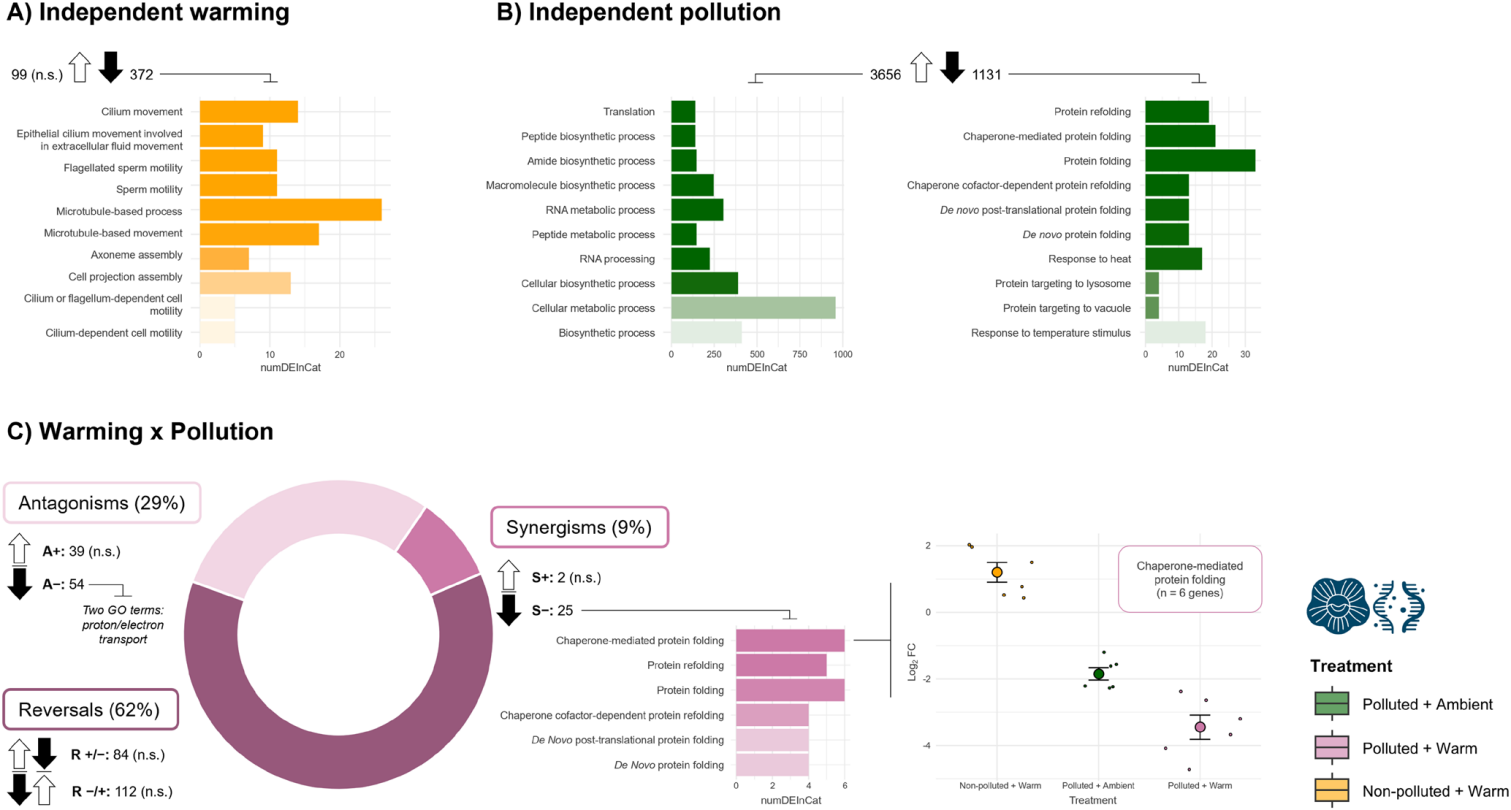
Barnacle transcriptomic responses at end of summer sampling period (September 2). Top enriched biological process (BP) Gene Ontology (GO) terms for individual genes significantly affected (*p*_adj_ < 0.05) by: (A) the main effect of warming; (B) the main effect of pollution; and (C) the warming × pollution interaction. Across all panels, arrows indicate the number and direction (up/downregulation) of differentially expressed individual genes, with ‘n.s.’ denoting cases where no collective GO term was significantly enriched (*p*_adj_ > 0.05), and bar plots showing top enriched GO terms for a given direction. In panel C (left), we show the classification of interaction types for genes with a significant interactive response (+/‐ indicating up‐ or down‐regulation respectively): (A+ = antagonistic positive; A− = antagonistic negative; S+ = synergistic positive; S− = synergistic negative; R+/− = reversal from positive to negative; R−/+ = reversal from negative to positive). The middle panel shows significant BP GO‐term enrichments for negative synergisms specifically, and the right panel shows mean (±SE) log_2_ fold‐change (FC) for individual genes within the top enriched BP GO term of these negative synergisms (GO term: Chaperone‐mediated protein folding; five HSP genes and one endoplasmic reticulum (ER) chaperone).

Exposure to warming alone induced a relatively weak transcriptomic response, significantly affecting the expression of 471 genes (*p*_adj_ < 0.05), of which nearly 80% were downregulated (372 genes) and significantly enriched for (i.e., selected for) cytoskeletal and structural (cilium‐associated) proteins (Figure 4A). In contrast, the polluted site was associated with a much stronger transcriptomic response, with significant changes in the expression of 4787 genes including 3656 upregulated genes (76%), largely associated with protein turnover and DNA repair. In the polluted site, downregulated genes were dominated by GO terms involved in protein folding (i.e., chaperone and proteostasis pathways; Figure 4B). There was a significant interactive effect of warming and pollution on the expression of 316 genes (Figure 4C). Reversals were the dominant interaction type by frequency (~62% of DEGs), followed by antagonisms (~29%) and limited synergisms (~9%). For antagonistic interactions, only downregulated genes displayed functional enrichment, with two GO terms significantly involved in electron/proton transport (e.g., COX genes). Synergistic upregulated genes showed no functional enrichment, whereas downregulated genes were significantly enriched for multiple protein folding GO terms. Within chaperone‐mediated protein folding (GO:0061077), five putative heat shock proteins (HSP) genes showed no significant induction in response to warming alone (*p*_adj_ > 0.05), but a significant downregulation in response to pollution (*p*_adj_ < 0.05), which was reinforced by their interaction (Figure 4C). No reversals showed any significant functional enrichment. Full DEG‐level outputs (log_2_FC, significance, database hits, interaction classifications) and GO enrichment outputs for all contrasts are provided in the accompanying workbooks, archived with the project data and code repository (see Data Availability).

### 3.2 Algal Responses

#### 3.2.1 Macroalgae Cover

**FIGURE 5:**
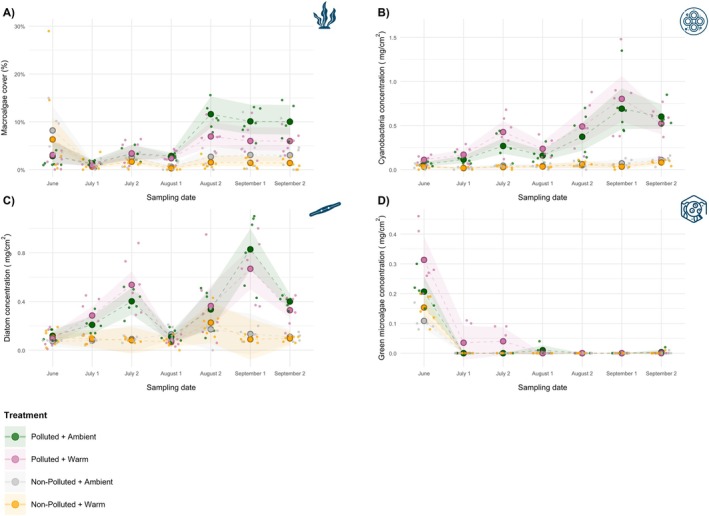
Algal responses to warming across polluted and non‐polluted sites on each sampling date. Large circles show GLM model‐estimated treatment means for each sampling date; semi‐transparent ribbons show 95% confidence intervals around those date‐specific estimates, with illustrative dashed connectors between treatment means (whole‐season temporal trends are evaluated with GAMMs; see SI Figure S10 for GAMM temporal visualisations). Smaller points are jittered raw replicate values to illustrate distributions around each estimated mean response. (A) Macroalgal cover (%). (B) Cyanobacteria concentration (mg/cm^2^). (C) Diatom concentration (mg/cm^2^). (D) Green microalgae concentration (mg/cm^2^; GLM fitted for June only; subsequent dates show empirical means with 95% CIs and raw data points). Full model specifications and diagnostics are provided in the SI (Table S3).

GLMs showed that macroalgal cover was higher at the polluted site on most dates, significantly so on all sampling dates from August 1 onwards (*β* ranging from 1.263 to 1.596, all *p* < 0.001). In contrast, warming tended to reduce cover with near‐significant effects (0.05 ≤ *p* < 0.10) observed in the latter half of the season (August 1, September 1, September 2; *β* ranging from −0.795 to −0.754) (Figure 5A; SI Table S7). The interaction between warming and pollution was neither significant nor consistent across dates (SI Table S7; Figure S6). Whole‐season GAMM predictions (adjusted *R* ^2^ = 0.655) corroborated the dominant seasonal pattern of higher macroalgal cover under pollution and lower cover under warming, with both main effects statistically significant (warming: *β* = −0.608, SE = 0.297, *p* = 0.041; pollution: *β* = 0.632, SE = 0.285, *p* = 0.027), while the interaction remained non‐significant (*β* = 0.317, SE = 0.410, *p* = 0.439) (SI Table S14; Figure S10D). Smooth terms were strongly significant across all treatments (all *p* < 0.001), with edf values (ranging from 2.451 to 4.102) indicating non‐linear seasonal fluctuations in each treatment combination (SI Table S14).

#### 3.2.2 Cyanobacteria Concentration

GLMs indicated that cyanobacteria concentration was higher at the polluted site on all dates from July 1 onwards, whereas warming showed no statistical significance (Figure 5B; SI Table S8). Significant positive interactions were observed in June (*β* = 0.057, SE = 0.027, *p* = 0.047) and July 2 (*β* = 0.175, SE = 0.080, *p* = 0.040), denoting synergistic effects on both dates (SI Table S8; Figure S7). In direct terms, these interactions indicate that cyanobacterial concentrations were higher than expected under the combined treatment on those dates; equivalently, the polluted‐site contrast was larger under warming on those dates. GAMM predictions (adjusted *R* ^2^ = 0.637) supported these findings: elevated seasonal concentration in the polluted site (*β* = 0.268, SE = 0.026, *p* < 0.001), whereas warming was non‐significant (*β* = −0.011, SE = 0.009, *p* = 0.188). Here, the interaction was positive and statistically significant (*β* = 0.086, SE = 0.039, *p* = 0.030), indicating a synergistic effect. However, the 95% CI overlaps our ±5% SESOI band (identity scale band = ±0.016; CI = [0.009, 0.162]); we therefore interpret this season‐wide pattern as a mild synergism. Smooth‐term results indicated significant seasonal variation across all four treatment combinations (SI Table S15).

#### 3.2.3 Diatom Concentration

Diatom concentration similarly reflected these patterns. GLMs showed that diatom concentration was positively and significantly influenced by pollution on the majority of dates, whereas warming only showed a significant negative effect on August 1 (*β* = −0.063, SE = 0.028, *p* = 0.033) (Figure 5C; SI Table S9). The interaction was inconsistent and insignificant across all sampling dates (SI Table S9; Figure S8). GAMM predictions (adjusted R^2^ = 0.779) supported a positive pollution effect (*β* = 0.230, SE = 0.047, *p* < 0.001), with warming remaining non‐significant (*β* = −0.004, SE = 0.047, *p* = 0.927). The interaction term similarly remained non‐significant (*β* = −0.003, SE = 0.066, *p* = 0.963) (SI Table S16; Figure S10F). Smooth term analysis indicated that temporal trends were highly significant in the polluted site (Ambient, polluted: edf = 5.777, *p* < 0.001; Warmed, polluted: edf = 5.818, p < 0.001), but were non‐significant in the non‐polluted site (SI Table S16).

#### 3.2.4 Green Microalgae Concentration

For green microalgae, only the June data provided sufficient variation to fit a GLM; on all subsequent sampling dates, concentrations were essentially zero. In June, the GLM indicated that green microalgae concentration was significantly higher in the polluted site (*β* = 0.098, SE = 0.028, *p* = 0.002). Similarly, the effect of warming also showed a positive association (*β* = 0.045, SE = 0.023, *p* = 0.062) (Figure 5D; SI Table S10). The interaction between warming and pollution was statistically insignificant (SI Table S10; Figure S9). Given near‐zero concentrations after June, no further GLMs or time‐series GAMM analyses were performed. Later time points are presented descriptively with raw data and means alongside the June GLM model predictions in Figure 5D.

## 4 Discussion

Our study reveals complex, temporally dynamic interactions between warming and ambient sewage‐associated pollution across two contrasting rocky shore sites. The prevalence of interactive responses across several response variables in our study echoes findings from several syntheses of multiple stressor studies across a variety of marine groups and life stages (Cabral et al. 2019; Gissi et al. 2021; Krishna et al. 2025; Przeslawski et al. 2015). By integrating functional‐group, population, trophic and transcriptomic responses, we show how local and global drivers interact across ecological scales, underscoring the need to quantify interactions when informing adaptive conservation and management in dynamic coastal zones.

### 4.1 Multi‐Level Invertebrate Responses

Consistent with our predictions, the contrast between the polluted and non‐polluted sites emerged as a dominant driver across several invertebrate responses at multiple levels of biological organisation. Barnacle abundance increased sharply at the polluted site throughout the season, similar to patterns reported in sewage‐impacted coastal systems (Courtenay et al. 2011). This marked increase at polluted sites suggests barnacle coverage could serve as an accessible visual indicator of water quality, particularly concerning sewage pollution. Furthermore, barnacle species are particularly sensitive to increasing temperatures, suffering from desiccation due to their sessile nature (Gedan et al. 2011; Kordas and Harley 2016; Lamb et al. 2014). Although the mean maximum temperature on black plates (38.1°C) did not exceed the thermal maximum for adult *Semibalanus balanoides* of ~42°C (Wethey 2002), it potentially surpassed juvenile barnacles' tolerance (Southward 1958), perhaps contributing to mortality or settlement difficulty during recruitment. While no evidence of increased metabolic processes or antioxidant activity was detected at the molecular level, this trend likely reflects a chronic transcriptional state following prolonged exposure throughout the season, as such responses are often transient and may return to a new steady‐state even when the stressor persists (López‐Maury et al. 2008). GLMs and whole‐season GAMMs consistently revealed positive interaction terms, with the exponentiated season‐long interaction indicating a ~66% increase in barnacle abundance over the null expectation. In direct ecological terms, warming reduced barnacle abundance at both sites, but this negative effect was weaker at the polluted site than at the non‐polluted site; equivalently, the polluted‐versus‐non‐polluted site contrast was larger under warming. Despite the effect of site pollution being so large independently, adding warming strengthened the effect beyond the null prediction, reflecting a mild synergism at this seasonal scale. This pollution dominance is mirrored at the molecular level, as the polluted site triggered a much larger transcriptional response than warming, and further demonstrated by synergistic interactions on chaperone activity that were driven by strong pollution‐associated downregulation. Similar masking has been observed in freshwater systems, where a stronger local stressor often masks regional climatic effects (Morris et al. 2022).

Barnacle body size exhibited complex temporal dynamics. In the early summer, body size was positively influenced at the polluted site on two sampling dates but later declined below those seen at the non‐polluted site (albeit statistically insignificant; see Figure 2B). Sewage‐impacted communities can show elevated secondary production and a higher proportion of smaller juveniles due to richer food supply (Cabral‐Oliveira et al. 2014), consistent with elevated barnacle abundance in polluted conditions. Higher densities may also intensify competition for space, favouring smaller individuals with lower energetic costs per unit area (Marchinko et al. 2004; Senthilnathan 2023). At the season level, the GAMM indicated that stressor main effects and their interaction were not significant, with patterns instead dominated by treatment‐specific temporal trajectories. Both non‐polluted treatments showed approximately linear increases through the season, whereas polluted treatments exhibited strongly non‐linear trajectories, consistent with fluctuating size structure through time under sewage exposure. Mean barnacle size can reflect a composite outcome of recruitment timing, growth and density dependence, with crowding and food supply influencing size distributions and reproductive thresholds rather than producing a single monotonic response (Giménez and Jenkins 2013; Leslie 2005; Scrosati and Freeman 2019; Scrosati and Holt 2021). Moreover, our size metric (rostro‐carnial plate measurements) is a morphological proxy that reflects integrated calcification, spacing of rostro‐carnial plates, and growth over preceding periods; as such, it may be less sensitive to rapid changes in soft‐tissue condition or short‐lived stress events (Burden et al. 2014; Mondal et al. 2021), reinforcing the interpretation of GAMM patterns as reflecting cohort/growth dynamics expressed over time.

Stable isotope analyses provide further mechanistic insights. At the polluted site, barnacle tissues exhibited significantly lower δ^15^N and δ^13^C values, indicating incorporation of sewage‐derived nitrogen and eutrophication‐enhanced carbon sources, respectively. Such effluent‐based nitrogen sources can often be isotopically lighter due to rapid microbial turnover, partial denitrification/nitrification, or the labile nature of nitrogen present in effluent (Dudley and Shima 2010; Laitano et al. 2018), then subsequently assimilated by filter‐feeding barnacles. This contrasts with the more typical expectation, where anthropogenic nutrient loads elevate δ^15^N via preferential δ^14^N loss during microbial processing (Costanzo et al. 2005; Savage 2005), indicating that the specific biochemical characteristics of the anthropogenic sewage effluent in our study may override conventional fractionation patterns. Likewise, a more negative δ^13^C in the polluted site indicates a shift in carbon sourcing, typically linked to eutrophication‐enhanced phytoplankton (Dudley and Shima 2010). In contrast, warming did not alter these isotopic signatures, supporting our assumption that the warming treatment effects were localised to the settlement plates. However, warming resulted in a significantly elevated C:N ratio. This increase reflects a physiological response, potentially involving a shift towards increased allocation of carbon‐rich compounds (e.g., lipids/carbohydrates) under thermal stress, while pollution did not alter this overall balance of assimilated nutrients (Zhang et al. 2018). Interestingly, our transcriptomic analyses are consistent with reduced investment in costly protein‐maintenance pathways, showing selective downregulation of protein‐folding genes in the polluted site, with this suppression further reinforced when warming and pollution co‐occur.

The molecular responses observed here contextualise findings at higher levels of biological organisation, while revealing physiological responses following chronic exposure to warming and pollution context. Warming alone induced a relatively weak transcriptional response, dominated by the downregulation of cilium‐ and cytoskeleton‐associated genes, potentially indicating cytoskeletal remodelling as a stress‐defence mechanism (Clark et al. 2018) or energy limitation associated with impaired feeding and growth (e.g., reduced suspension‐feeding efficiency) (Humphries 2013). In contrast, the polluted site induced extensive transcriptional regulation, including upregulation of protein‐turnover and DNA‐repair pathways, consistent with proteotoxic and genotoxic stress typical of exposure to xenobiotics (foreign contaminants) in aquatic invertebrates inhabiting sewage‐impacted environments (Freitas et al. 2023). These patterns suggest that contaminants and associated microbial by‐products co‐discharged with sewage, such as bacterial proliferation and their toxins, may contribute to the observed responses alongside nutrient enrichment (Albini et al. 2023), despite our field contrast being defined by a categorical site difference in ambient sewage‐associated nutrient conditions rather than a replicated pollution treatment. Consistently higher cyanobacterial concentrations at our polluted site further support the possibility that microbially‐derived cyanotoxins form part of this co‐pollutant mixture. A clear pattern was the strong downregulation of protein‐folding and chaperone genes in the polluted site, which was further intensified under combined warming. Because protein‐synthesis pathways were not similarly suppressed, this may indicate a selective alteration of proteostasis. One possibility is energetic reprioritisation towards rapid growth at the expense of inducible stress‐resistance pathways, a trade‐off proposed for organisms under chronic stress (López‐Maury et al. 2008). Alternatively, contaminant‐induced interference with HSP regulation, or long‐term acclimation upon chronic exposure, may contribute to dampened inducible HSP responses, as reported for intertidal crustaceans (Collins, Clark, et al. 2021; Collins, Peck, and Clark 2021). Overall, it is likely that contaminants associated with the sewage discharge impose regulatory effects not captured by nutrient data alone, while the ecological responses we observe still reflect a realistic sewage‐pollution context in which nutrient enrichment and co‐occurring contaminants are tightly coupled.

The nature of the interactions observed at the molecular level differed from those observed at higher levels of biological organisation, with most classified as reversals. Such reversals are likely because transcriptomic data capture responses across a wide dynamic range and large number of response variables (Brasseur et al. 2022), enabling large, sign‐changing interaction effects that are more constrained in organismal or ecological traits. Gene expression can also exhibit rapid, compensatory changes that override the direction of single‐stressor effects before these responses are buffered into higher‐level phenotypes. Although previous multiple stressor transcriptomic studies typically report antagonisms (Brasseur et al. 2022; Delnat et al. 2020), reversals are rarely defined in molecular analyses, and many reversals detected here would have been classified as antagonistic under earlier frameworks, consistent with the existing literature. Low annotation rates prevented a detailed mechanistic interpretation, but the dominance of reversal and antagonistic interactions suggests that ambient pollution substantially reshapes thermal responses at the molecular level, partly buffering ecological impacts of warming while potentially compromising proteostasis in exposed cells.

Grazer occurrence was generally higher at the polluted site, with enrichment effects statistically supported on two of seven sampling dates. Previous work has shown mixed gastropod responses to elevated nutrients, with abundance increasing up to a nitrate threshold before declining (Conde et al. 2020). In our study, grazers exhibited notably higher occurrence at the polluted site compared to the non‐polluted site, potentially due to eutrophication supporting local primary producer proliferation as a food source (Kraufvelin et al. 2002). Elevated barnacle abundance may also have enhanced grazer settlement in polluted areas, by acting as biological facilitators and providing microhabitats, as has been observed with littorinid periwinkles (Silva et al. 2015), common in our research area. A sharp decline in grazer occurrence was observed on August 1, coinciding with high precipitation and sewage effluent release events in the area (see Supplementary Methods (Analyses); SI Figure S11), possibly reflecting threshold‐exceeding nutrients and turbidity (Cabral‐Oliveira et al. 2014; Conde et al. 2020). Grazer occurrence recovered despite continued effluent discharges, perhaps due to temporary refuge or movement given motility. Additional factors such as effluent composition, unmonitored pollution events, or high footfall prior to this sampling date may have influenced this pattern. Furthermore, difficulties in model convergence owing to the high proportion of zero counts necessitated our choice of binary models (see SI, Appendix S2), hindering ecological interpretability and nuance of findings. Moreover, at the seasonal scale, the GAMM corroborated a strong positive pollution effect on grazer occurrence, while warming and the interaction remained non‐significant; temporal structure was only detectable in a subset of treatment combinations, consistent with more episodic dynamics rather than a uniform seasonal trajectory.

### 4.2 Functional‐Group Algal Responses

Algal functional groups responded strongly to nutrient pollution, with distinct patterns among macroalgae, cyanobacteria and diatoms. GLMs indicated macroalgal cover increased significantly at the polluted site from August 1 onward. Elevated nitrate levels commonly drive macroalgal proliferation across many temperate coastal systems, and particularly in relatively nutrient‐poor settings, including species with distinct seasonal growth patterns such as *Saccorhiza polyschides* , which is common in our sampling region (Colvard and Helmuth 2017; Maúre et al. 2021; Wheeler and Björnsäter 1992). Greater barnacle abundance at the polluted site may also have increased small‐scale surface heterogeneity and propagule retention, potentially facilitating macroalgal settlement, although this interpretation remains tentative as barnacle cover was not quantified directly (Steneck et al. 2002). Whole‐season GAMMs confirmed the positive influence of site‐level pollution and warming's significant negative effect, consistent with thermal constraints on macroalgal physiology (Román et al. 2020). In addition to warming, solar ultraviolet radiation (UVR) exposure can stimulate enzymatic activities, increase the repair rate of photosystems and enhance photosynthesis (Ji et al. 2016; Ji and Gao 2021), perhaps explaining initially higher coverage across all treatments. However, prolonged exposure can lead to mortality, especially when temperature thresholds are exceeded (Fernández et al. 2015; Vye et al. 2015; Zou and Gao 2014), corroborated in our study, as near‐significant warming only induced reductions in late summer. Moreover, macroalgal abundance is tightly coupled with grazing effects of gastropods and abiotic factors (Jonsson et al. 2006; Schiel et al. 2004), which perhaps regulated excessive growth across all treatments.

Cyanobacteria concentration was consistently and significantly elevated at the polluted site from July 1 onward, while warming showed no independent effect. Interestingly, two discrete GLMs (June, July 2) detected significant positive interactions between warming and pollution context, suggesting transient, date‐specific synergistic effects. In plain terms, cyanobacterial concentrations were higher than expected under the combined treatment on those dates, meaning that the polluted‐versus‐non‐polluted site contrast became larger under warming. At the seasonal scale, the GAMM confirmed site‐level pollution as the primary driver; the positive interaction was statistically significant and indicative of a mild synergism under our ±5% criterion, suggesting a modest amplification. This synergism echoes research concluding that nutrient enrichment and warming often synergistically increase growth rate of cyanobacteria (Lürling et al. 2017; Rigosi et al. 2014); however, it is noted that this effect varies substantially among taxa (Rigosi et al. 2014). Moreover, the significant smooth terms capturing marked temporal fluctuations across treatments underscore the importance of evaluating stressor interactions across a range of temporal resolutions. Given cyanobacterial toxicity and implications for bathing water quality, these dynamics, together with evidence for cyclic shifts in biofilm composition under sewage effluent (Jaubet et al. 2024) reinforce that nutrient reduction is likely to yield the greatest mitigation of harmful blooms.

Diatoms displayed similar nutrient‐driven increases, with the effect of site‐level pollution significant on most dates and concentration rising through summer. This suggests nutrient pollution as the primary driver of diatom proliferation, potentially via phosphate‐favoured, pollution‐tolerant species, consistent with other studies (Blanco 2024; Nunes et al. 2024; Rahman et al. 2024). This trend was notably perturbed, however, with a significant negative impact of warming on August 1. Similar patterns were observed in other algal groups, and possibly indicate that responses are subject to additional abiotic influences such as co‐occurring heavy metal contamination, otherwise unaccounted for in the scope of this study. The interaction between warming and pollution was inconsistent and statistically insignificant across both discrete sampling dates and across the season. Notably however, smooth term analysis revealed that temporal trends in diatom abundance were highly significant in the polluted site, whereas no significant temporal trends were observed in non‐polluted sites. This mirrors our grazer findings, whereby environmental variability associated with pollution events may have induced temporal fluctuations in concentration.

Green microalgae showed a starkly ephemeral response, with concentrations reaching near‐absence following June. This contrasts strongly with other algal groups, which showed more consistent elevation in the polluted site. The June GLM nonetheless showed a positive pollution effect, consistent with eutrophication's initial boost to photosynthetic organisms (Cabral‐Oliveira et al. 2014). Furthermore, with increasing and sustained thermal stress and solar radiation, concentrations showed sharp declines, aligning with the idea that moderate warming can enhance photosynthesis to a threshold, but cumulative thermal and UV stress can abruptly override early advantages provided by both warming and pollution context (Zou and Gao 2014).

### 4.3 Implications and Future Directions

Our findings emphasise the importance of temporal and multi‐scale approaches for understanding multiple‐stressor dynamics on rocky shores, spanning molecular to functional‐group‐level responses. Longer‐term experiments that track adaptation and recovery under different stressor regimes are needed to separate transient from persistent responses and to inform predictive models that reflect dynamic interactions (Jackson et al. 2021; Vos et al. 2023). In our experiment, plates were installed before the first summer survey; therefore, responses observed here likely integrate not only summer conditions but also earlier treatment effects on biofilm development, settlement, early survival and subsequent seasonal dynamics (see Supplementary Methods (Experimental)). A combined strategy integrating laboratory experiments, transcriptomics and other ‘omics’ tools, computational modelling and mesocosm/field studies will be critical for developing data‐driven models of how global and local stressors interact over extended periods and across levels of biological organisation.

Strengthening the evidence base on ecological and molecular effects can also support efforts to address sewage pollution impacts in coastal waters, improving accountability and informing actionable initiatives such as the Storm Overflows Discharge Reduction Plan in the UK, alongside stronger enforcement by regulators to prosecute ongoing offences (Department for Environment, Food, and Rural Affairs 2023). Future studies should extend beyond nutrient gradients to include co‐occurring stressors such as pCO_2_, silicates, heavy metals and microbially derived toxins, providing a more complete view of variable sewage composition against a backdrop of global change. Where feasible, future field designs should also expand spatial replication of contrasting site pollution contexts while preserving comparability in shore type, tidal height, hydrodynamics and pollution footprint independence.

In studying stressor interactions, traditional analyses often rely on additive, linear models, where deviations indicate non‐linearity (Pirotta et al. 2022; Schäfer and Piggott 2018). However, this method oversimplifies the complexity of natural stressor‐response dynamics (Schäfer et al. 2023). Statistical interactions depend not only on stressor intensities and biology, but also on the statistical process and null model employed (Duncan and Kefford 2021; Mack et al. 2022). Accordingly, interaction classifications should be interpreted as model‐based effect modification on a defined scale, and complemented with effect sizes, uncertainty and biological context when drawing mechanistic conclusions (Schäfer et al. 2023; Spake et al. 2023).

Managing the combined effects of warming, sewage pollution and other stressors in intertidal ecosystems is inherently complex. Stressors create distinct local footprints, yet ecosystem‐level responses are shaped by context, biogeography and spatial extent (Low et al. 2023). Our experiment provides a cost‐effective, scalable multiple‐stressor approach that can be deployed more widely to test context dependence. Moreover, addressing the geographic skew in marine multiple stressor research, with a dominance of ‘Global North’ studies (Gissi et al. 2021) will also require fostering collaborations and capacity with researchers in diverse regions, particularly in the ‘Global South’ (Dahdouh‐Guebas et al. 2003; Tolochko and Vadrot 2021), to generate geographically diverse datasets spanning different shore types, climatic regimes, land‐use settings and pollution contexts for rocky shore management across spatiotemporal scales.

## 5 Conclusions

To our knowledge, this is the first rocky shore field experiment to combine experimental warming within a local, ambient site contrast in sewage‐associated pollution, quantifying responses across multiple levels of ecosystem organisation: from gene expression to functional‐group‐level structure. The outcomes of this work contribute towards advancing and justifying further multiple stressor research on marine and specifically intertidal ecosystems, alongside tangible management insights such as barnacle abundance as an effective visual indicator of intertidal water body health, as well as confirmation of both periodic and seasonal synergisms seen in toxic cyanobacteria. This study is particularly timely in the UK, adding to the evidence base of academic knowledge supporting sanctions and stronger regulation of privatised water companies to reduce harmful sewage inputs nationwide. The feasibility of our in situ method should encourage informed, practical management and mitigation of both local and global stressors, with strong potential to catalyse international research and collaboration.

## Author Contributions

**Ramesh Wilson:** conceptualization (equal), data curation (lead), formal analysis (lead), funding acquisition (lead), investigation (lead), methodology (lead), project administration (lead), software (lead), validation (lead), visualization (lead), writing – original draft (lead), writing – review and editing (lead). **Katie Driver:** data curation (supporting), investigation (lead), writing – original draft (supporting), writing – review and editing (supporting). **James Orr:** formal analysis (supporting), investigation (supporting), methodology (supporting), software (supporting), writing – original draft (supporting), writing – review and editing (supporting). **Manuela Truebano:** data curation (lead), formal analysis (lead), software (lead), validation (lead), writing – original draft (supporting), writing – review and editing (supporting). **Michael Collins:** data curation (lead), formal analysis (lead), software (lead), validation (lead), visualization (lead), writing – original draft (supporting), writing – review and editing (supporting). **Michelle Jackson:** conceptualization (equal), data curation (supporting), formal analysis (supporting), investigation (supporting), methodology (supporting), supervision (lead), writing – original draft (supporting), writing – review and editing (supporting).

## Funding

This work was supported by Natural Environment Research Council (NERC) Oxford Doctoral Training Partnership in Environmental Research (NE/S007474/1), Pembroke College (University of Oxford), the Eurofins Foundation and the Challenger Society for Marine Science.

## Ethics Statement

No animal ethics approval was sought as this study did not involve vertebrates, and it did not involve regulated ‘protected animals’ under common legal frameworks governing animal research. In the UK, the Animals (Scientific Procedures) Act 1986 applies to living vertebrates and living cephalopods as protected animals, while EU Directive 2010/63/EU similarly applies to live non‐human vertebrates and live cephalopods. The focal taxa in this study (barnacles, non‐cephalopod gastropods, macroalgae and microphytobenthos) fall outside those scopes. The study also did not involve captivity, invasive procedures, or deliberate harm beyond exposure to natural field conditions and passive settlement‐plate manipulation. Permission for field installation was granted by Natural England and the Crown Estate.

## Conflicts of Interest

The authors declare no conflicts of interest.

## Supporting information


**Data S1:** ece373368‐sup‐0001‐DataS1.zip.

## Data Availability

The ecological datasets and analysis code supporting this study, including RNA‐seq annotated differential‐expression and GO enrichment output workbooks, are archived at Zenodo (https://doi.org/10.5281/zenodo.18121767) and maintained in the associated GitHub repository. Raw transcriptome sequencing data are available via ArrayExpress (Accession: E‐MTAB‐16541). Public sewage release records are available via Southern Water's Rivers and Seas Watch portal (Newhaven outfall; https://experience.arcgis.com/experience/e9a1db8d193d4cd582d550285a3aeb44/page/Map/). Precipitation time series used for SI Figure S11 were downloaded from Visual Crossing and are not redistributed in the public repository as per third‐party terms; users can obtain equivalent raw data directly from Visual Crossing (https://www.visualcrossing.com/weather‐query‐builder/).
