# Supplementary material for: Gene‐to‐Population Level Responses to Multiple Stressors on the Rocky Shore
Source: Ecol Evol. 2026 Apr 7;16(4):e73368. doi: 10.1002/ece3.73368 (PMC13055495; doi:10.1002/ece3.73368)
Supplement: Supplementary file 1 — Data S1: ece373368‐sup‐0001‐DataS1.zip. [file ECE3-16-e73368-s001.zip › ece373368-sup-0003-SupplementaryInformation2.docx]

**Supplementary Information 2: Pad Construction**

**Gene-to-population level responses to multiple stressors on the rocky shore**

**Contents:**

1. **Plate specifications**
2. **Logger installation**
3. **Epoxy surface application**

These building instructions are to be used as a guide. **Materials and availability will vary on availability, but are permissible provided the dimensions, colour difference, and sizes of materials are the same. Example suppliers have been provided and highlighted in green and underlined throughout**, but if the same piece cannot be sourced, ensure the specifications and quality are matched.


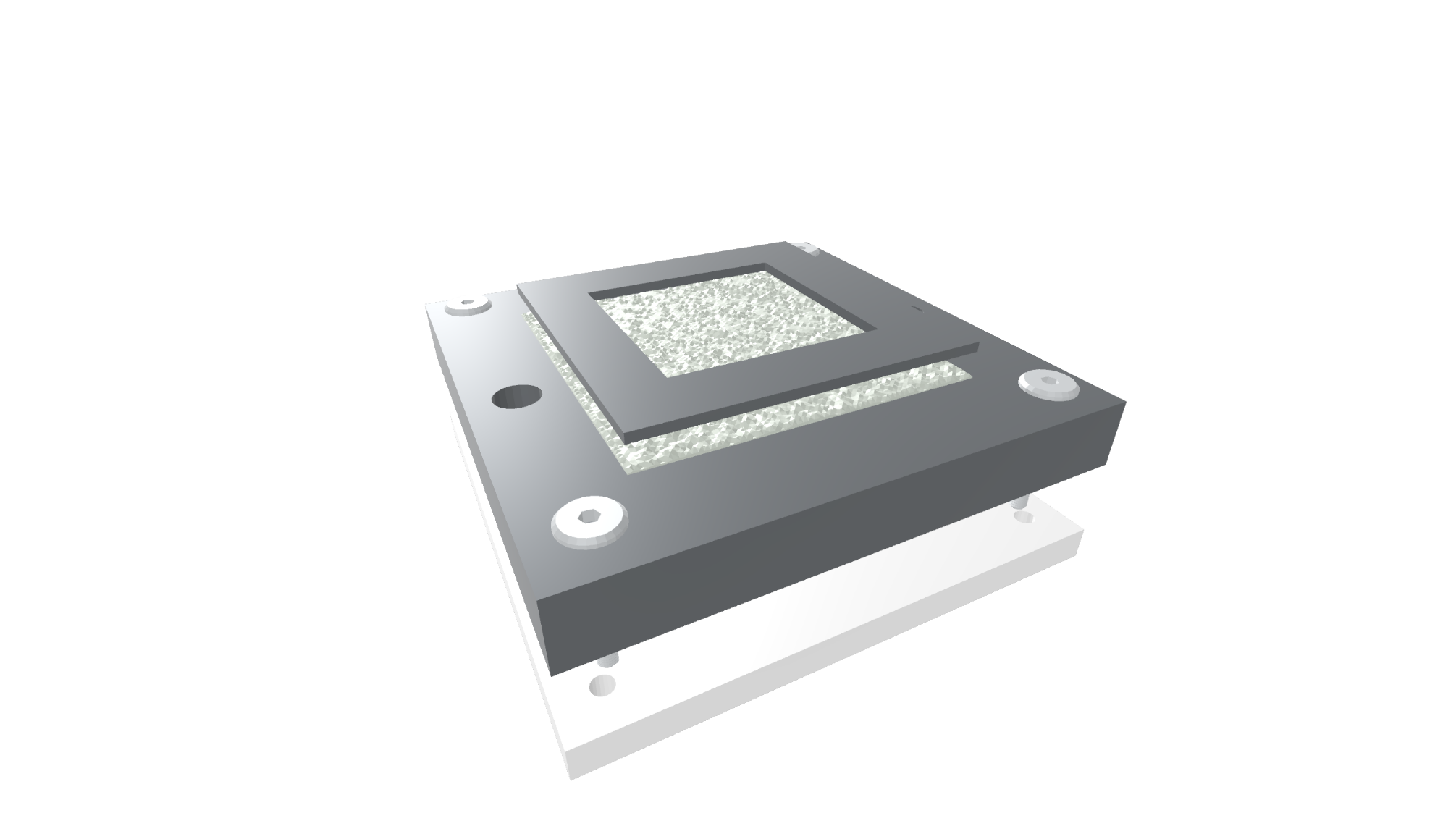


**1. Plate specifications**

Values given are in millimetres (mm). Visual dimensions not to scale. Note**, measurements with an asterisk ‘*’ are customisable to accommodate the dimension of the logger subsequently embedded.**

*HDPE plastic squares should be sourced following the below specifications:*

- **Face (top) squares**: 160mm x 160mm x 20mm: 12 black, and 12 white (following experimental method employed in this paper)
- **Back (bottom) squares**: 160mm x 160mm x 10mm: 24 (following experimental method; colour does not matter, and the number must match the total number of face squares)
- **Suggested UK provider: Bay Plastics.**
  - <https://www.plasticstockist.com/HDPE-Sheet-PE300.aspx>

*
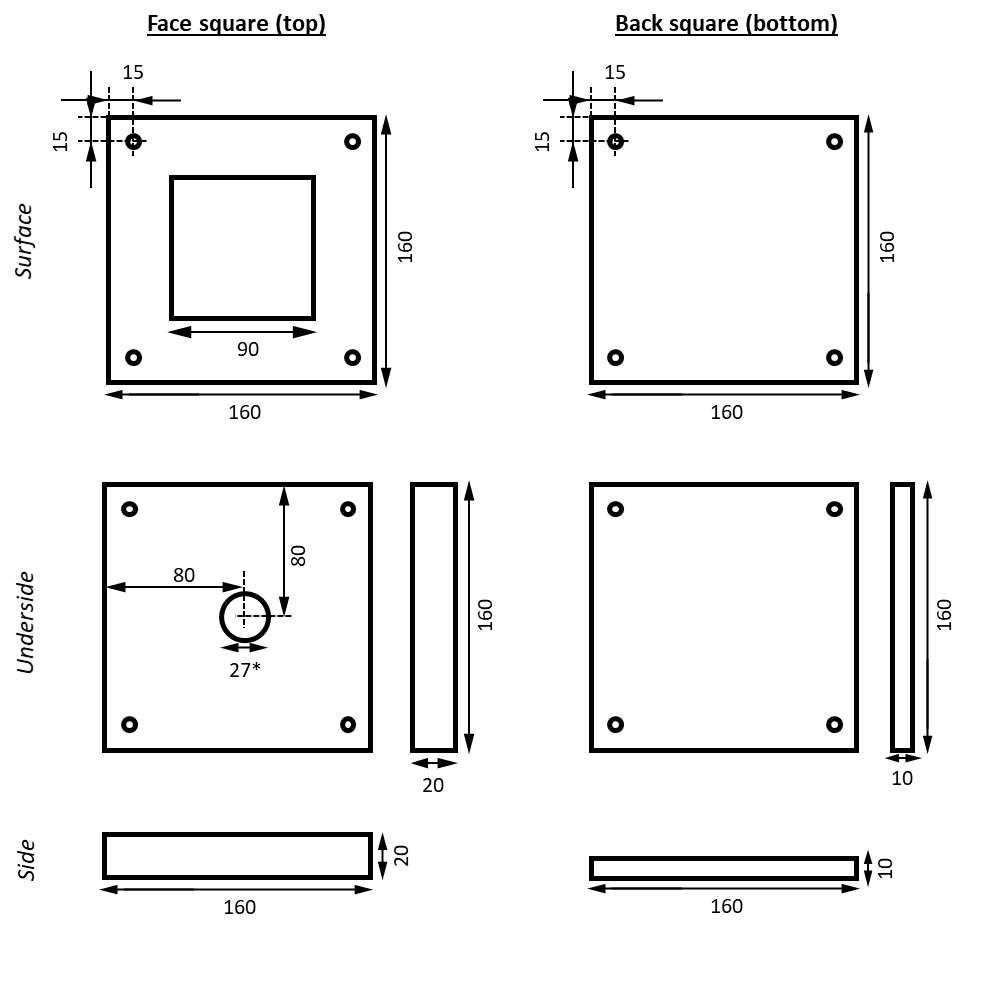
*

**2. Logger installation**

**The temperature logger will be placed into the underside of the face plate,** sandwiched between the face and back plates.

- **Suggested temperature loggers: EnvLogger T2.4 (27mm).**
  - <https://electricblue.eu/temperature-envloggers>

**Create a niche for the temperature logger to sit in the centre of the face plate, so that it lies flat.** To do so, **there are two options:**


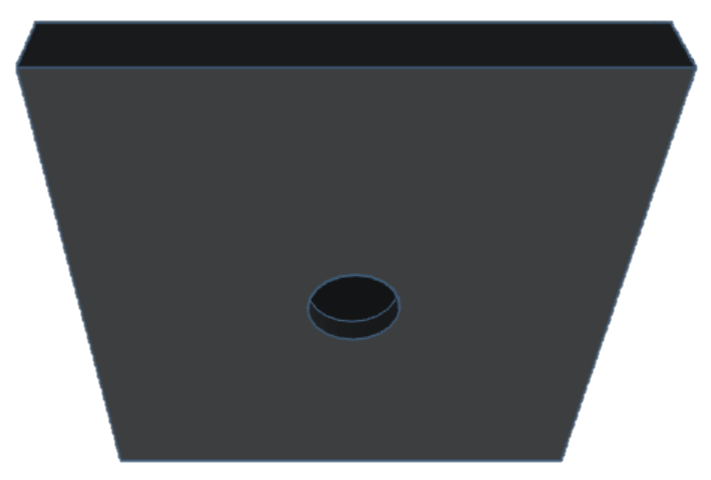


1. **Manually drill the circular niche** into the underside of the face square, using an appropriately sized flat drill bit (e.g., a 27mm flat drill bit for a temperature logger of 27mm diameter as employed in this study).

- Must ensure that the depth of the recession is only as deep as needed to accommodate the logger (e.g., 11mm depth for loggers used in this study); drilling deeper risks going through the plate, and will not ensure the logger is flush.

1.
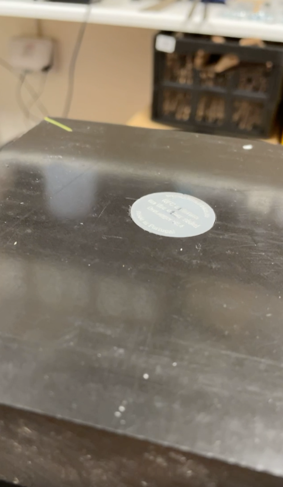
**Use a CNC machine to consistently and accurately drill** the recession with the exact measurements to accommodate the logger.

- This method was employed in this study, by outsourcing to the same retailer that provided the HDPE plastic at an additional fee (Bay Plastics).
- This method is preferable for consistency and logistical ease, however poses more financial cost.

**Next, we then clamp the face and back plate** together; this will ensure they are **properly aligned to create holes to drill and secure them together**, eventually with the logger sandwiched between.

1.
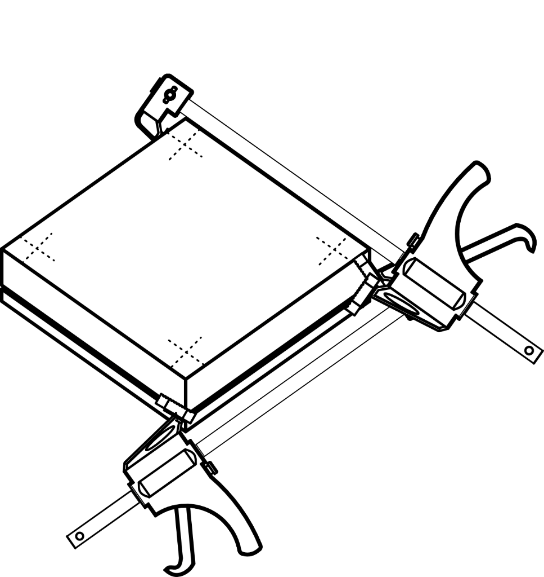
**The bolt holes need to be created to attach the face plate to the back plate.**

- Clamp the plates together using at least two quick clamps, or g-clamps.
- Measure approximately 15mm both ways from each corner of the plate, and draw lines 90° from the edges until they cross.
- Using a 6mm drill bit, drill through both plates fully.
  - If using a hand drill, there is likely to be slight variation between each hole drilled; the face and back squares should always align with the correct drill holes. Etching a pattern/number into one of the sides of both the face, and the back plates is recommended, to ensure consistent square pairs and orientation.
  - *Tip: slide an M6 bolt into the holes as they are drilled to prevent sliding while subsequent holes are drilled.*

1. **Embed the temperature logger**

-
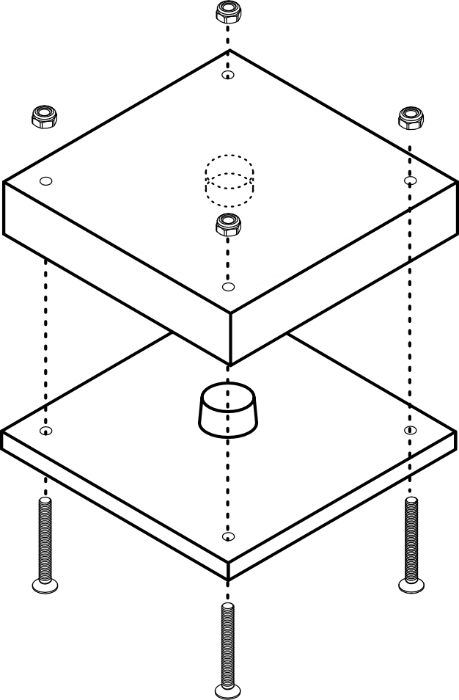
Ensure that the logger is set up to the recording/logging parameters you desire, and is logging correctly (depending on the recording range of the logger, you can calibrate either before, or after installation; ensure you check this prior).
- Place the logger in the niche in the underside of the face square, with the sensor against the top of the face plate.
- Insert M6 50mm bolts into the predrilled holes from the back of the back square, through to the face square, so that the M6 bolts can be tightened on the face plate side using a spanner (this is to ensure that the back plate can lie flat on the installation surface, without protrusion from bolts).
  - **Suggested bolts: M6 Hexagon Nylon Locking Nuts.**
    - <https://www.accu.co.uk/hexagon-nylon-locking-nuts/7948-HNN-M6-A2>
  - **Suggested screws: M6 x 50mm Socket Countersunk Screws.**
    - <https://www.accu.co.uk/countersunk-socket-head-screws/5492-SSK-M6-50-A2>

**3. Epoxy surface application**

Now you will need to attach an **epoxy surface to the front plate**. This is the **actual experimental, colonisation region, which will be texturised in order to simulate natural substrate.**


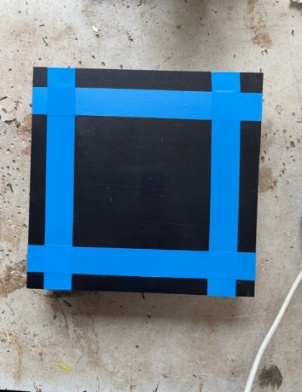

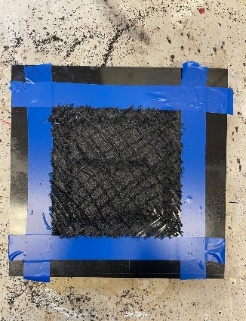


1. **Mark out the area using masking/painter’s tape.**

- 9cm x 9xm in the centre (following the area employed in this study).

1. **Texturise/roughen the 9cm square in order to ensure the epoxy will adequately stick**, and not peel off (this is a very likely possibility if not texturised).

- Can cross hatch texturise using a blade/scalpel, or a sanding hand tool (e.g., Dremel rotary multitool).

1. Mix the epoxy and **apply onto the square within the area of the masking tape border**, ensuring layer is not too thick (no more than ~5mm; the thicker, the more likely to peel off).

- Apply quickly, depending on the cure time.
- **Suggested brand: PC-Products 080115 PC-11 Marine Grade Paste Epoxy**
  - <https://pcepoxy.co.uk/products/pc-11-paste-epoxy>


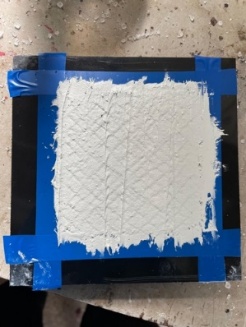

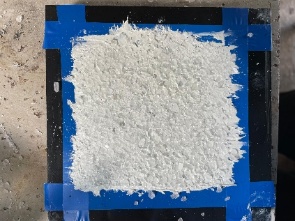


1. **Apply rock salt and push into the surface** of the wet epoxy; this will create the bumpy texture for settlement.
2. Before the epoxy dries completely, **remove the tape.**
3. When the epoxy has completely cured**, rinse the surface of the plates with boiling water thoroughly to remove the rock salt.**
4.
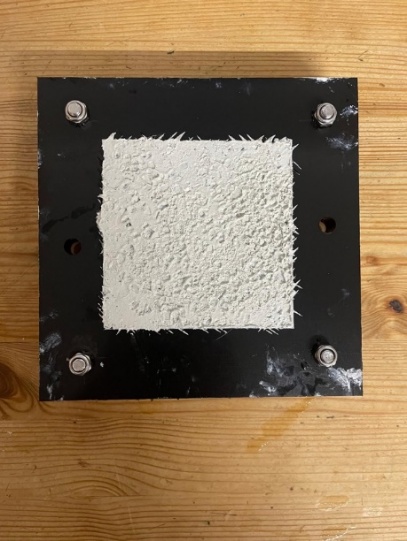
**With your completed plate, drill a hole on the left and right of the epoxy square;** this is where the screw will go through to attach onto the substrate (recommended size, 8mm diameter hole).
