# Supplementary material for: Gene‐to‐Population Level Responses to Multiple Stressors on the Rocky Shore
Source: Ecol Evol. 2026 Apr 7;16(4):e73368. doi: 10.1002/ece3.73368 (PMC13055495; doi:10.1002/ece3.73368)
Supplement: Supplementary file 1 — Data S1: ece373368‐sup‐0001‐DataS1.zip. [file ECE3-16-e73368-s001.zip › ece373368-sup-0002-SupplementaryInformation1.docx]

**Supplementary Information 1**

**Gene-to-population level responses to multiple stressors on the rocky shore**

**Contents:**

- **Supplementary Methods: Experimental**
  - Experimental verifications
  - Settlement plate design
  - Automated counting
- **Supplementary Methods: Analyses**
  - Grazer model convergence
  - GLM interaction conditional plots
  - GAMM model specifications
  - Local precipitation and effluent release events
- **Supplementary Tables**
  - Methods
  - Results
- **Supplementary Figures**
  - Methods
  - Results
- **Supplementary References**

**Supplementary Methods: Experimental**

**Experimental verifications**

Site and nutrient pollution verification

The polluted replicates were installed at Friar’s Bay (50.785° N, 0.021° E). This region receives sewage inputs from several major sewage overflow systems, including the Seaford storm overflow discharge valve (5.4 km east) as well as the Newhaven long sea outfall (2 km SE offshore) (Figure S1). Such concerns make Peacehaven an even more ideal location study sewage pollution effects in this experiment. We hypothesised that more pollution would accumulate at Friar’s Bay than at Bastion Steps (1.36 km to the west; 50.789° N, -0.002° E), due to more sewage overflows localised around the site, the enclosed shape of Newhaven Bay, and the eastward littoral drift of the Sussex coast, concentrating effluent plumes away from the Bastion Steps (see Figure S1 for regional map).

Nutrient pollution levels were verified before installation via three pre-installation water quality checks of nitrate and phosphate, with the polluted site showing consistently higher measurements on each date (Table S1). Furthermore, on each sampling date, nitrate and phosphate readings were taken at both the polluted and non-polluted site to continually verify pollution levels. Five samples at each location were taken from the surrounding area (each roughly 5 m from the installation site in different directions) and averaged (Figure S2). Nitrate was recorded using an *in situ* Hanna colorimetry multiprobe (HI-782) and phosphate using a portable Hanna photometer testing kit (HI-97717).

During the experiment, the nutrient-polluted site showed consistently higher levels of nitrate and phosphate relative to the non-polluted site (Figure S2). Together with our pre-installation verification values, provided confidence in our categorical site selection.

Scope of site-level pollution contrast

Because only one shore represented each pollution category, the pollution term in this experimental design is not spatially replicated. We therefore interpret it as an ambient site-context contrast between a more sewage-impacted shore (Friar’s Bay) and a less impacted shore (Bastion Steps), supported by repeated nutrient verifications. This inferential limit is important in dictating warming as the experimentally replicated manipulation within each site, whereas sewage-associated pollution reflects the ambient context of the selected shores. In designing the field study, we prioritised close geographic proximity, comparable shore type, and spatially discrete site contexts so as to minimise broader differences in climate, exposure regime, and other local environmental conditions that would otherwise confound inferences. Increasing the number of sites would have improved nominal replication of the ambient pollution contrast, but would also have required greater spatial extent and introduced additional uncontrolled heterogeneity among shores, while making it harder to maintain independence and avoid overlap in sewage influence footprints. Practical constraints associated with permitting and maintaining comparable *in situ* installations further limited the number of feasible sites. Accordingly, site-associated pollution effects are interpreted cautiously, and repeated nutrient verification is used to support site designation rather than to imply full replication of an ambient pollution treatment.

Nitrate sensor limitations and use of proportional elevation

For nitrate, we used a nitrate ion-selective electrode (ISE) on the multiparameter probe as a relative indicator of enrichment between sites, not as an absolute concentration measurement. Nitrate ISEs are susceptible to matrix interference effects in saline waters, with chloride/salinity capable of introducing substantial positive bias, and so the absolute nitrate values in Table S1 should be treated as qualitative only.

Because salinity was effectively the same at the polluted and non-polluted sites (36.08 vs 36.30 PSU across the experimental period) and paired samples were taken within minutes on each verification date, any salinity-linked bias is expected to be shared within a date, making within-date proportional differences interpretable. We therefore report and use only the proportional elevation in the nitrate-ISE signal at the polluted site relative to the non-polluted control (expressed as a ratio and plotted as % increase in Fig. S2 and Fig. 1). This enrichment signal is additionally supported by phosphate measurements from a dedicated photometric method (Table S1), which is not subject to the same nitrate-ISE chloride interference. Together, these nutrient data underpin our categorical designation of Friar’s Bay as ‘polluted’ and Bastion Steps as ‘non-polluted’ for the factorial analyses.

Warming verification

Temperature loggers embedded within plates were configured to take measurements every 90 min at a 0.1 °C resolution for the duration of the study from installation, using ElectricBlue EnvLogger T2.4 loggers (27 mm hard-acrylic format; see manufacturer technical documentation: <https://img1.wsimg.com/blobby/go/62cbf3ca-7d0d-4afa-9ded-6866ac65ded2/downloads/EnvLogger_documentation_package_compressed.pdf?ver=1772534277634>).

Across the entire period, mean max values (i.e., maximum value recorded on each plate, averaged across treatment replicates) showed a difference of +1.6 °C (38.0 °C and 36.4 °C for black and white plates respectively). Additionally, during the summer period, when including only readings from days when low tide occurred during peak daytime hours, the mean average daily maximum (mean ADM; calculated as the residual of each plate’s ADM from the grand mean on each sampling date, then averaged across treatment replicates), showed a warming increase of +0.8 °C in the warmed treatment. Additional temperature differences can be seen in Table S2. While a categorical difference in warming is evident, EnvLoggers were houses in a hard and inert acrylic resin, potentially confounding the accuracy of temperature readings when embedded within an additional layer of HDPE plastic. Future iterations of the method may better capture differences in warming by using thinner plate surface layers, and considering different logger encasement options, as both may have diminished the true magnitude of recorded temperature differences.

**Settlement plate design**

In many intertidal organisms, body temperature is more directly correlated with substrate as opposed to atmospheric temperatures, as the heat budget is more dominated by conduction than convection (Denny & Harley, 2006). Warmed settlement plates, which raise body temperatures via conduction, have been employed in multiple studies (Kordas et al., 2015, 2017; Kordas & Harley, 2016; LaScala‐Gruenewald & Denny, 2020), demonstrating the effectiveness of the method; particularly for organisms with high surface area in contact with substratum, such as barnacles, limpets, and prostrate algae.

Settlement plates were made of 3 cm thick black or white High-Density Polyethylene (HDPE) plastic squares (2 cm for treatment surface, affixed on top of an additional 1 cm white square). Each plate was 16 cm x 16 cm, topped with a centred 9 cm x 9 cm area of pale epoxy (PC-Products^®^; PC-11 Marine Grade Epoxy, Off White), roughly 3 mm thick. Our design was inspired by Kordas et al. (2015), with edits including expansion of surface epoxy size, logger type, and thickness of surface squares to accommodate the logger. The surfaces of plates were textured prior to epoxy application to ensure a strong bond, as it otherwise peeled off within a few months of installation. To create the heterogeneous settlement surface, we pressed a single layer of rock salt into the epoxy, which was dissolved prior to installation. The underside of the top plate was drilled using a CNC machine to create a cylindrical recess the size of the ElectricBlue EnvLogger. This allowed the EnvLogger to be sandwiched between the plates, to avoid protruding hardware, which may have otherwise created additional surface heterogeneity and encouraged biased settlement.

Settlement place installation geometry

Plates were mounted horizontally (i.e., approximately 0° relative to the local substrate), with the epoxy settlement surface facing directly upward. At both sites they were fixed to the upper faces of near-horizontal artificial substrata extending seaward from the shore. Because the biologically active settlement surface was horizontal, a compass-facing orientation in the same sense as a vertical panel was not directly applicable; instead, consistency was achieved by standardising installation height and using comparable upper horizontal surfaces across sites. The full plate assembly was approximately 3 cm thick, meaning the settlement surface stood slightly above the immediately surrounding substratum. This design is particularly suited to sessile recruits and surface-associated taxa, but responses of motile grazers should be interpreted as occupancy and use of the settlement surface rather than exhaustive abundance on the surrounding shore.

**Field establishment period and interpretation**

Plates were installed in January 2023 and left *in situ* until the first summer survey in June 2023 to permit natural biofilm development, settlement, and early community establishment under continuous treatment exposure. We avoid the term ‘acclimatisation’ here because the pre-survey interval was not a separate pre-treatment phase; rather, it formed the initial portion of the continuous experimental exposure history. Consequently, any treatment-linked differences in spring colonisation or early development are considered part of the cumulative seasonal response measured in this study, not an uncontrolled bias arising prior to the summer experiment. All plates were installed simultaneously, at the same shore height within site, and were only compared among synchronously occurring treatment groups, thereby standardising exposure history within the design. This approach is consistent with passive warming settlement-tile studies that install substrates before or at the onset of the main summer exposure period (Kordas et al., 2015, 2017; Kordas & Harley, 2016).

**Automated counting**

To standardise barnacle abundance counts across plates, treatments, and sampling dates, we used the CountThings mobile app workflow to analyse overhead photographs taken within the 40% stencil window to avoid edge effects (see Methods). CountThings operates via “Counting Templates” that detect and count target objects in images, and was configured for our system using manually annotated images collected during the field-establishment period and early sampling. Training images deliberately spanned multiple barnacle morphologies, sizes (new recruits to adults), lighting conditions, epibiont cover, and epoxy textures, so that the detector would generalise across the full range of field image conditions. An additional label was used to distinguish live individuals (with an intact operculum visible) from dead individuals (operculum absent). We applied a single decision threshold across the project to balance precision and recall, and maintained quality-control measures: 1) plates with very low densities (≤10 individuals) were counted *in situ* by eye rather than by model; and 2) images showing prohibitive motion blur, glare, or water droplets were entered as NA in the dataframe and excluded. Model performance used standard metrics: precision, recall, F1 score, mean average precision (mAP), intersection-over-union (IoU), average precision per class, average recall, and false-positive rate. Detailed model architecture and internal optimisation/augmentation settings are not user-exposed as CountThings deploys templates within a proprietary environment ([www.countthings.com](http://www.countthings.com)).

**RNA-seq sampling timing and interpretation**

RNA-seq sampling was conducted once at the end of summer to characterise the integrated late-season transcriptional state associated with repeated warming and pollution exposure across the experimental season. It was not designed to isolate an acute minute-to-hour heat-shock response to a single low-tide event. By the time of sampling, individuals had experienced months of repeated tidal emersion cycles on their assigned plates; differential expression is therefore interpreted primarily as a late-season transcriptomic phenotype reflecting sustained exposure, acclimatory state, and/or baseline expression differences among treatment contexts. Because samples were collected during the same field session across treatments, the immediate sampling context was standardised, but, as with any single-time-point field transcriptome, acute and chronic components cannot be separated completely. We therefore interpret these RNA-seq data as an integrated end-of-season transcriptional snapshot and discuss them accordingly (López-Maury et al., 2008; Sleight et al., 2018).

**Supplementary Methods: Analyses**

**Grazer model convergence**

Our grazer data presented statistical challenges due to the high proportion of zeros in the dataset, combined with sporadic occurrences of high counts on some sampling dates. These data characteristics led to complete separation issues, where certain predictor levels were perfectly associated with a single outcome (e.g., total grazer absence under specific conditions). In such cases, standard logistic regression struggles, as maximum likelihood estimation inflates coefficient estimates to extreme values, causing unreliable inference and misleading predicted probabilities.

**We initially explored count-based formulations for grazer abundance, including Poisson, negative binomial, zero-inflated, and hurdle-type models. However, the combination of extremely sparse positive counts and very high zero frequencies led to unstable fits, poor convergence, and weak identification of separate zero-generating and count-generating processes, particularly within individual sampling dates. In addition, two-component formulations reduced ecological interpretability for this dataset, because the central biological question was whether grazers were using settlement surfaces at all, rather than precise variation in positive counts once present.**

To address this, bias-reduced logistic regression (BRGLM) was used via the brglm2 package (Kosmidis & Firth, 2021). This approach applies penalty-based shrinkage to coefficient estimates, preventing overfitting and producing more accurate probability estimates. By reducing the inflation of extreme values, bias-reduced methods improve the stability and interpretability of models. This adjustment was crucial in ensuring that grazer presence probabilities were not artificially skewed due to numerical artifacts from standard logistic regression. Similarly, for the whole-season time-series analysis, we fitted a binomial logit GAMM, with stability aided by GAMM penalisation and REML estimation with select = TRUE, allowing additional shrinkage/selection of smooth terms while retaining predictions on the probability scale via the logit link.

**GLM interaction conditional plots**

Conditional plots provide a visual representation of how predicted response variables change under different combinations of interacting predictors. These plots are particularly useful for assessing effect modification and asymmetric responses, offering a clear visual assessment of whether stressor interactions amplify, mitigate, or shift expected outcomes (Spake et al., 2023). A fundamental advantage of conditional plots is their ability to reveal context dependence, i.e., how the effect of one predictor (e.g., warming) varies depending on the level of another predictor (e.g., pollution). In these plots, the x-axis represents one categorical stressor (here, the warming treatment), while the y-axis represents the predicted response variable (on the appropriate model scale). The two lines within each panel indicate predicted responses under different levels of the second categorical stressor (pollution), with shaded confidence bands. Panels correspond to different sampling dates and GLMs, with interaction significance provided.

**GAMM model specifications**

For each response variable, a generalised additive mixed model (GAMM) was fitted to capture season-long trajectories of stressor effects. These models were used because responses were sampled repeatedly across the summer and were not assumed to change linearly through time; smooth functions allow the data to determine whether temporal patterns are approximately linear or non-linear.

Time was represented as Days_Since_Start (days since the first sampling date). To allow temporal dynamics to differ among treatments, we fitted separate smooths for each warming × pollution combination using a factor-by smooth of the form (Days_Since_Start, by = interaction(Pad_Colour, Pad_Region), k = 7). The basis dimension was set to k = 7 to match the seven sampling dates, providing sufficient capacity to represent seasonal structure without pre-specifying a particular trajectory (Wood, 2025). We did not fix the smooth degrees of freedom: instead, the effective degrees of freedom (EDF) for each smooth were estimated from the data under the model’s smoothing penalty (i.e., we did not use the argument fx = TRUE). This allows each response variable and each treatment-specific smoothing term to retain as much or as little temporal variability as warranted, ensuring consistency across responses while avoiding arbitrary constraints or overfitting on temporal complexity. In model outputs, the EDF indicates the complexity of the fitted temporal pattern: values near 1 correspond to an approximately linear trend through time, whereas larger values indicate increasing curvature. A significant smooth term indicates that the response varies with time within that treatment combination (i.e., the fitted smooth is not compatible with a flat, time-invariant effect). Notably, a smooth can be significant even when EDF ≈ 1, which reflects evidence for a non-zero linear temporal trend rather than complex non-linearity.

Models were fitted by restricted maximum likelihood (REML). To account for repeated measures of the same settlement plate across sampling dates, plate identity (Pad_ID) was included as a random effect using s(Pad_ID, bs = "re"). We refer to these models as GAMMs because they include both smooth terms and a random effect, although they were fitted using mgcv::gam() rather than gamm(), with the random effect implemented as a penalised random-effect smooth. Diagnostic evaluations were conducted to assess residual normality and heteroscedasticity, ensuring model assumptions were met.

**Local precipitation and effluent release events**

We compared the intensity of local precipitation in Peacehaven with the duration of storm overflow sewage release events to assess whether there was an association, which may partially explain observed declines in several groups (notably grazers, barnacle size, and cyanobacteria). This was particularly relevant for observed declines in multiple group responses between July 2-August 1, which corresponded to high precipitation events and high durations of sewage effluent release events between this period.

Effluent release data were gathered from Southern Water’s public repository service, ‘Rivers and Seas Watch’, and precipitation data was gathered from the Visual Crossing API service for the Peacehaven area. Notably, for effluent release data, we only included releases from the Newhaven Outfall (see Figure S1). This was due to outfall monitor failures reported in other outfalls in the area, as reported in the Environment Agency Event Duration Monitoring - Storm Overflows: Annual Returns, between 2022 and at least August 2023. Therefore, while Newhaven Outfall is included here, this is a conservative representative of the true frequency or duration of effluent releases in the area.

**Supplementary Tables**

**Methods**

Experimental verifications

| **Month** | **Site** | **Nitrate ISE readings (NO^3-^ mg/l, uncorrected)** | **PO_4_^3-^ readings (mg/)** |
| --- | --- | --- | --- |
| September 2022 | Polluted | 61.2; 60.1; 56.8 (x̄ = **59.37**) | 0.12; 0.10; 0.10 (x̄ = **0.110**) |
|  | Non-polluted | 24.8; 29.9; 28.8 (x̄ = **27.83**) | 0.00; 0.00; 0.00 (x̄ = **0.000**) |
| October 2022 | Polluted | 66.0; 64.8; 65.0 (x̄ = **65.27**) | 0.10; 0.06; 0.18 (x̄ = **0.113**) |
|  | Non-polluted | 28.9; 34.7; 30.1 (x̄ = **31.23**) | 0.00; 0.01; 0.00 (x̄ = **0.003**) |
| November 2022 | Polluted | 68.1; 65.1; 62.2 (x̄ = **65.13**) | 0.20; 0.11; 0.12 (x̄ = **0.143**) |
|  | Non-polluted | 37.7; 32.1; 30.3 (x̄ = **33.37**) | 0.00; 0.00; 0.00 (x̄ = **0.000**) |

**Table S1**. Pre-experiment nitrate and phosphate verification readings for site selection. Three verification excursions were completed (September, October, November 2022) prior to installation. At each date and site, three samples were taken and averaged (means shown in the main text, Fig. 1D). Nitrate ISE readings (NO^3-^ mg/l; uncorrected) are shown only as the raw instrument response and are not interpreted as absolute seawater nitrate concentrations due to chloride interference. Phosphate values are absolute concentrations from the photometer (PO_4_^3-^).

| **Treatment** | **Mean max. (raw):**  Installation and sampling | **Mean ADM:**  Installation and sampling | **Mean ADM:**  Sampling only | **Tide-adjusted mean ADM:**  Sampling only |
| --- | --- | --- | --- | --- |
| **Black** | 38.0 | 17.6 | 25.5 | 29.5 |
| **White** | 36.4 | 17.4 | 25.2 | 28.7 |

**Table S2:** Summary of recorded plate temperatures. Mean max represents the maximum values ever recorded by plate loggers, averaged across all replicates in each warming group. Mean average daily max (ADM) values were calculated by using the residual of the ADM for each plate logger from the grand mean on each sampling date, subsequently averaged across all replicates in each warming group. This was further tide-adjusted, by only including days in which low tide occurred during peak daytime hours (defined for simplicity here as 10:00-14:00), thereby maximising exposure and thermal absorption.

Model choices and diagnostics

| **Response variable** | **Model type** | **Model scale** | **Notes/diagnostics** |
| --- | --- | --- | --- |
| **Barnacle abundance** | GLMs: Negative binomial  GAMM: Negative binomial | Multiplicative (link = ‘log’) | Discrete count data, right-skewed, and overdispersed.  Whole-season GAMM includes separate smooth of time per treatment, plus pad random effect to account for repeated measures. |
| **Barnacle size** | GLMs: Gaussian  GAMM: Gaussian | Additive (link = ‘identity) | Continuous, normally distributed on raw scale.  Whole-season GAMM includes separate smooth of time per treatment, plus pad random effect to account for repeated measures. |
| **Grazer presence** | GLMs: Bias-reduced binomial logistic  GAMM: Binomial logistic | Multiplicative on the odds scale (link = ‘logit’) | Presence/absence data with high proportion of 0s, with (quasi-)separation risk. Per-date fits use bias reduction (brglmFit) to stabilise estimates and avoid infinite/unstable coefficients.  Whole-season GAMM includes separate smooth of time per treatment, plus pad random effect to account for repeated measures. Model further includes select = TRUE argument which adds shrinkage penalties to smooth terms so unsupported effects can be driven towards zero (i.e., performing smooth-term selection and stabilising the fit), as required to ensure successful convergence. |
| **Macroalgae cover** | GLMs: Beta regression  GAMM: Beta regression | Multiplicative on the odds scale (link = ‘logit’) | Treated as proportional (bounded 0-1) with asymmetric distributions.  Whole-season GAMM includes separate smooth of time per treatment, plus pad random effect to account for repeated measures. |
| **Cyanobacteria concentration** | GLMs: Tweedie  GAMM: Tweedie | Additive (link = ‘identity) | Continuous, non-negative, often with many 0s. Per-date GLMs fix power to default 1.5 for stability/consistency, allowing data with a point mass at zero and a continuous distribution, without adding constants.  Whole-season GAMM includes separate smooth of time per treatment, plus pad random effect to account for repeated measures. |
| **Diatom concentration** | GLMs: Gaussian  GAMM: Gaussian | Additive (link = ‘identity) | Continuous, normally distributed on the raw scale.  Whole-season GAMM includes separate smooth of time per treatment, plus pad random effect to account for repeated measures. |
| **Green microalgae concentration** | GLM (June): Gamma  GAMM: NA | Additive (link = ‘identity) | Continuous, non-negative, right-skewed on the raw scale.  No GAMM constructed due to near zero values across all treatments following June; therefore, no additional time points statistically modelled/analysed. |

**Table S3:** Model choices and diagnostics for both GLMs and GAMMs. Residuals, diagnostic plots, and model comparisons available in additional analysis R scripts.

**Results**

GLM model summaries

| **Date** | **Stressor** | **Estimate** | **SE** | **p-value** | **Δadd** | **Δobs** | **Exponentiated null prediction** | **Exponentiated observed value** | **Exponentiated interaction ratio** | **Interaction ratio 95% CI** | **Classification** |
| --- | --- | --- | --- | --- | --- | --- | --- | --- | --- | --- | --- |
| June | Warming | -0.693 | 0.149 | <0.001*** | 1.394 | 1.808 | 161.250 | 243.830 | 1.512 | [1.060, 2.162] | Synergism |
|  | Pollution | 2.087 | 0.120 | <0.001*** |  |  |  |  |  |  |  |
|  | Interaction | 0.414 | 0.182 | 0.023* |  |  |  |  |  |  |  |
| July 1 | Warming | -0.790 | 0.168 | <0.001*** | 1.222 | 1.723 | 147.050 | 242.670 | 1.650 | [1.089, 2.504] | Synergism |
|  | Pollution | 2.012 | 0.142 | <0.001*** |  |  |  |  |  |  |  |
|  | Interaction | 0.501 | 0.212 | 0.018* |  |  |  |  |  |  |  |
| July 2 | Warming | -0.767 | 0.170 | <0.001*** | 1.243 | 1.636 | 146.650 | 217.330 | 1.482 | [0.972, 2.264] | NA |
|  | Pollution | 2.009 | 0.145 | <0.001*** |  |  |  |  |  |  |  |
|  | Interaction | 0.393 | 0.216 | 0.068. |  |  |  |  |  |  |  |
| August 1 | Warming | -0.849 | 0.178 | <0.001*** | 1.178 | 1.659 | 139.110 | 225.000 | 1.618 | [1.038, 2.524] | Synergism (within ±5% 'additive' boundary) |
|  | Pollution | 2.026 | 0.152 | <0.001*** |  |  |  |  |  |  |  |
|  | Interaction | 0.481 | 0.227 | 0.034* |  |  |  |  |  |  |  |
| August 2 | Warming | -0.961 | 0.177 | <0.001*** | 1.044 | 1.579 | 124.970 | 213.330 | 1.707 | [1.102, 2.651] | Synergism |
|  | Pollution | 2.005 | 0.148 | <0.001*** |  |  |  |  |  |  |  |
|  | Interaction | 0.535 | 0.224 | 0.017* |  |  |  |  |  |  |  |
| September 1 | Warming | -0.963 | 0.183 | <0.001*** | 1.046 | 1.592 | 124.300 | 214.670 | 1.727 | [1.095, 2.729] | Synergism |
|  | Pollution | 2.009 | 0.155 | <0.001*** |  |  |  |  |  |  |  |
|  | Interaction | 0.546 | 0.233 | 0.019* |  |  |  |  |  |  |  |
| September 2 | Warming | -0.980 | 0.181 | <0.001*** | 1.012 | 1.568 | 123.340 | 215.000 | 1.743 | [1.111, 2.741] | Synergism |
|  | Pollution | 1.992 | 0.153 | <0.001*** |  |  |  |  |  |  |  |
|  | Interaction | 0.556 | 0.230 | 0.016* |  |  |  |  |  |  |  |

**Table S4:** Barnacle abundance GLMs (log scale).

| **Date** | **Stressor** | **Estimate** | **SE** | **p-value** | **Δadd** | **Δobs** | **Null prediction** | **Observed value** | **±5% band** | **Interaction 95% CI** | **Classification** |
| --- | --- | --- | --- | --- | --- | --- | --- | --- | --- | --- | --- |
| June | Warming | -0.054 | 0.167 | 0.752 | -0.05 | 0.171 | 2.763 | 2.984 | ±0.138 | [-0.243, 0.685] | NA |
|  | Pollution | 0.003 | 0.167 | 0.984 |  |  |  |  |  |  |  |
|  | Interaction | 0.221 | 0.237 | 0.361 |  |  |  |  |  |  |  |
| July 1 | Warming | -0.126 | 0.140 | 0.376 | 0.236 | 0.445 | 3.043 | 3.252 | ±0.152 | [-0.178, 0.597] | NA |
|  | Pollution | 0.362 | 0.140 | 0.017* |  |  |  |  |  |  |  |
|  | Interaction | 0.209 | 0.198 | 0.302 |  |  |  |  |  |  |  |
| July 2 | Warming | -0.096 | 0.154 | 0.540 | 0.318 | 0.169 | 3.248 | 3.100 | ±0.162 | [-0.575, 0.278] | NA |
|  | Pollution | 0.414 | 0.154 | 0.014* |  |  |  |  |  |  |  |
|  | Interaction | -0.148 | 0.218 | 0.504 |  |  |  |  |  |  |  |
| August 1 | Warming | -0.232 | 0.155 | 0.149 | -0.520 | -0.295 | 2.585 | 2.810 | ±0.129 | [-0.204, 0.654] | NA |
|  | Pollution | -0.288 | 0.155 | 0.078. |  |  |  |  |  |  |  |
|  | Interaction | 0.225 | 0.219 | 0.316 |  |  |  |  |  |  |  |
| August 2 | Warming | -0.300 | 0.176 | 0.105 | -0.591 | -0.280 | 2.652 | 2.964 | ±0.133 | [-0.177, 0.800] | NA |
|  | Pollution | -0.292 | 0.176 | 0.114 |  |  |  |  |  |  |  |
|  | Interaction | 0.312 | 0.249 | 0.226 |  |  |  |  |  |  |  |
| September 1 | Warming | -0.229 | 0.169 | 0.188 | -0.578 | -0.205 | 2.550 | 2.923 | ±0.127 | [-0.094, 0.840] | NA |
|  | Pollution | -0.349 | 0.169 | 0.052. |  |  |  |  |  |  |  |
|  | Interaction | 0.373 | 0.238 | 0.133 |  |  |  |  |  |  |  |
| September 2 | Warming | -0.238 | 0.223 | 0.300 | -0.431 | -0.246 | 2.911 | 3.096 | ±0.146 | [-0.435, 0.804] | NA |
|  | Pollution | -0.193 | 0.223 | 0.397 |  |  |  |  |  |  |  |
|  | Interaction | 0.185 | 0.316 | 0.566 |  |  |  |  |  |  |  |

**Table S5:** Barnacle size GLMs (Gaussian, identity scale).

| **Date** | **Stressor** | **Estimate** | **SE** | **p-value** | **Δadd** | **Δobs** | **Null probability** | **Observed probability** | **Interaction odds ratio** | **Interaction ratio 95% CI** | **Classification** |
| --- | --- | --- | --- | --- | --- | --- | --- | --- | --- | --- | --- |
| June | Warming | 0.000 | 2.242 | 1.000 | 3.864 | 3.864 | 0.786 | 0.786 | 1.000 | [0.006, 179.037] | NA |
|  | Pollution | 3.864 | 1.872 | 0.039* |  |  |  |  |  |  |  |
|  | Interaction | 0.000 | 2.647 | 1.000 |  |  |  |  |  |  |  |
| July 1 | Warming | 0.000 | 2.242 | 1.000 | 3.153 | 5.13 | 0.643 | 0.929 | 7.222 | [0.026, 2021.585] | NA |
|  | Pollution | 3.153 | 1.800 | 0.080. |  |  |  |  |  |  |  |
|  | Interaction | 1.977 | 2.875 | 0.492 |  |  |  |  |  |  |  |
| July 2 | Warming | 0.000 | 2.242 | 1.000 | 3.864 | 3.153 | 0.786 | 0.643 | 0.491 | [0.003, 79.632] | NA |
|  | Pollution | 3.864 | 1.872 | 0.039* |  |  |  |  |  |  |  |
|  | Interaction | -0.712 | 2.596 | 0.784 |  |  |  |  |  |  |  |
| August 1 | Warming | -1.977 | 1.800 | 0.272 | -3.954 | -1.977 | 0.011 | 0.071 | 7.222 | [0.026, 2021.585] | NA |
|  | Pollution | -1.977 | 1.800 | 0.272 |  |  |  |  |  |  |  |
|  | Interaction | 1.977 | 2.875 | 0.492 |  |  |  |  |  |  |  |
| August 2 | Warming | -1.266 | 1.872 | 0.499 | 1.333 | 2.599 | 0.508 | 0.786 | 3.545 | [0.036, 348.932] | NA |
|  | Pollution | 2.599 | 1.407 | 0.065. |  |  |  |  |  |  |  |
|  | Interaction | 1.266 | 2.342 | 0.589 |  |  |  |  |  |  |  |
| September 1 | Warming | 0.000 | 1.407 | 1.000 | 1.299 | 3.864 | 0.500 | 0.929 | 13.000 | [0.152, 1115.238] | NA |
|  | Pollution | 1.299 | 1.287 | 0.313 |  |  |  |  |  |  |  |
|  | Interaction | 2.565 | 2.271 | 0.259 |  |  |  |  |  |  |  |
| September 2 | Warming | -1.977 | 1.800 | 0.272 | 1.176 | 3.153 | 0.643 | 0.929 | 7.222 | [0.026, 2021.585] | NA |
|  | Pollution | 3.153 | 1.800 | 0.080. |  |  |  |  |  |  |  |
|  | Interaction | 1.977 | 2.875 | 0.492 |  |  |  |  |  |  |  |

**Table S6:** Grazer presence probability GLMs (logit scale).

| **Date** | **Stressor** | **Estimate** | **SE** | **p-value** | **Δadd** | **Δobs** | **Null proportion** | **Observed proportion** | **Odds ratio** | **Interaction ratio 95% CI** | **Classification** |
| --- | --- | --- | --- | --- | --- | --- | --- | --- | --- | --- | --- |
| June | Warming | -0.280 | 0.399 | 0.483 | -1.398 | -1.037 | 0.022 | 0.031 | 1.435 | [0.409, 5.032] | NA |
|  | Pollution | -1.118 | 0.456 | 0.014* |  |  |  |  |  |  |  |
|  | Interaction | 0.361 | 0.640 | 0.573 |  |  |  |  |  |  |  |
| July 1 | Warming | -0.520 | 0.410 | 0.204 | -0.286 | -0.129 | 0.008 | 0.009 | 1.170 | [0.399, 3.428] | NA |
|  | Pollution | 0.234 | 0.358 | 0.513 |  |  |  |  |  |  |  |
|  | Interaction | 0.157 | 0.549 | 0.775 |  |  |  |  |  |  |  |
| July 2 | Warming | -0.460 | 0.386 | 0.233 | -0.204 | 0.273 | 0.021 | 0.034 | 1.611 | [0.603, 4.303] | NA |
|  | Pollution | 0.256 | 0.338 | 0.449 |  |  |  |  |  |  |  |
|  | Interaction | 0.476 | 0.502 | 0.342 |  |  |  |  |  |  |  |
| August 1 | Warming | -0.754 | 0.392 | 0.055. | 0.843 | 1.406 | 0.014 | 0.024 | 1.757 | [0.757, 4.080] | NA |
|  | Pollution | 1.596 | 0.270 | <0.001*** |  |  |  |  |  |  |  |
|  | Interaction | 0.564 | 0.430 | 0.190 |  |  |  |  |  |  |  |
| August 2 | Warming | -0.582 | 0.438 | 0.185 | 0.974 | 0.991 | 0.068 | 0.069 | 1.017 | [0.377, 2.746] | NA |
|  | Pollution | 1.555 | 0.332 | <0.001*** |  |  |  |  |  |  |  |
|  | Interaction | 0.017 | 0.507 | 0.974 |  |  |  |  |  |  |  |
| September 1 | Warming | -0.788 | 0.436 | 0.071. | 0.475 | 0.696 | 0.049 | 0.060 | 1.248 | [0.458, 3.401] | NA |
|  | Pollution | 1.263 | 0.323 | <0.001*** |  |  |  |  |  |  |  |
|  | Interaction | 0.221 | 0.512 | 0.666 |  |  |  |  |  |  |  |
| September 2 | Warming | -0.795 | 0.439 | 0.070. | 0.479 | 0.714 | 0.048 | 0.060 | 1.265 | [0.462, 3.463] | NA |
|  | Pollution | 1.274 | 0.325 | <0.001*** |  |  |  |  |  |  |  |
|  | Interaction | 0.235 | 0.514 | 0.648 |  |  |  |  |  |  |  |

**Table S7:** Macroalgae cover GLMs (logit scale).

| **Date** | **Stressor** | **Estimate** | **SE** | **p-value** | **Δadd** | **Δobs** | **Null prediction** | **Observed value** | **Interaction ratio 95% CI** | **±5% band** | **Classification** |
| --- | --- | --- | --- | --- | --- | --- | --- | --- | --- | --- | --- |
| June | Warming | -0.005 | 0.012 | 0.677 | 0.018 | 0.075 | 0.055 | 0.112 | [0.006, 0.114] | ±0.003 | Synergism |
|  | Pollution | 0.023 | 0.016 | 0.148 |  |  |  |  |  |  |  |
|  | Interaction | 0.057 | 0.027 | 0.047* |  |  |  |  |  |  |  |
| July 1 | Warming | 0.002 | 0.008 | 0.832 | 0.100 | 0.153 | 0.117 | 0.170 | [-0.021, 0.135] | ±0.006 | NA |
|  | Pollution | 0.098 | 0.023 | <0.001*** |  |  |  |  |  |  |  |
|  | Interaction | 0.053 | 0.038 | 0.180 |  |  |  |  |  |  |  |
| July 2 | Warming | -0.017 | 0.015 | 0.264 | 0.207 | 0.382 | 0.252 | 0.427 | [0.022, 0.343] | ±0.013 | Synergism |
|  | Pollution | 0.223 | 0.047 | <0.001*** |  |  |  |  |  |  |  |
|  | Interaction | 0.175 | 0.080 | 0.040* |  |  |  |  |  |  |  |
| August 1 | Warming | -0.007 | 0.015 | 0.657 | 0.112 | 0.195 | 0.153 | 0.237 | [-0.020, 0.195] | ±0.008 | NA |
|  | Pollution | 0.118 | 0.033 | 0.002 ** |  |  |  |  |  |  |  |
|  | Interaction | 0.083 | 0.053 | 0.132 |  |  |  |  |  |  |  |
| August 2 | Warming | 0.012 | 0.028 | 0.676 | 0.335 | 0.440 | 0.385 | 0.490 | [-0.155, 0.382] | ±0.019 | NA |
|  | Pollution | 0.323 | 0.083 | <0.001*** |  |  |  |  |  |  |  |
|  | Interaction | 0.105 | 0.131 | 0.431 |  |  |  |  |  |  |  |
| September 1 | Warming | -0.038 | 0.025 | 0.138 | 0.582 | 0.732 | 0.653 | 0.803 | [-0.207, 0.519] | ±0.033 | NA |
|  | Pollution | 0.620 | 0.120 | <0.001*** |  |  |  |  |  |  |  |
|  | Interaction | 0.150 | 0.179 | 0.413 |  |  |  |  |  |  |  |
| September 2 | Warming | -0.028 | 0.025 | 0.271 | 0.463 | 0.413 | 0.573 | 0.523 | [-0.245, 0.142] | ±0.029 | NA |
|  | Pollution | 0.492 | 0.073 | <0.001*** |  |  |  |  |  |  |  |
|  | Interaction | -0.050 | 0.097 | 0.613 |  |  |  |  |  |  |  |

**Table S8:** Cyanobacteria concentration GLMs (Tweedie, identity scale).

| **Date** | **Stressor** | **Estimate** | **SE** | **p-value** | **Δadd** | **Δobs** | **Null prediction** | **Observed value** | **Interaction ratio 95% CI** | **±5% band** | **Classification** |
| --- | --- | --- | --- | --- | --- | --- | --- | --- | --- | --- | --- |
| June | Warming | 0.032 | 0.032 | 0.327 | 0.077 | 0.022 | 0.150 | 0.095 | [-0.142, 0.032] | ±0.008 | NA |
|  | Pollution | 0.045 | 0.032 | 0.169 |  |  |  |  |  |  |  |
|  | Interaction | -0.055 | 0.045 | 0.231 |  |  |  |  |  |  |  |
| July 1 | Warming | 0.013 | 0.049 | 0.788 | 0.142 | 0.205 | 0.222 | 0.285 | [-0.073, 0.199] | ±0.011 | NA |
|  | Pollution | 0.128 | 0.049 | 0.017* |  |  |  |  |  |  |  |
|  | Interaction | 0.063 | 0.069 | 0.372 |  |  |  |  |  |  |  |
| July 2 | Warming | -0.010 | 0.074 | 0.894 | 0.300 | 0.445 | 0.392 | 0.537 | [-0.061, 0.351] | ±0.020 | NA |
|  | Pollution | 0.310 | 0.074 | <0.001*** |  |  |  |  |  |  |  |
|  | Interaction | 0.145 | 0.105 | 0.183 |  |  |  |  |  |  |  |
| August 1 | Warming | -0.063 | 0.028 | 0.033* | -0.083 | -0.057 | 0.047 | 0.073 | [-0.050, 0.103] | ±0.002 | NA |
|  | Pollution | -0.020 | 0.028 | 0.479 |  |  |  |  |  |  |  |
|  | Interaction | 0.027 | 0.039 | 0.504 |  |  |  |  |  |  |  |
| August 2 | Warming | 0.055 | 0.115 | 0.637 | 0.218 | 0.192 | 0.390 | 0.363 | [-0.345, 0.291] | ±0.020 | NA |
|  | Pollution | 0.163 | 0.115 | 0.170 |  |  |  |  |  |  |  |
|  | Interaction | -0.027 | 0.162 | 0.871 |  |  |  |  |  |  |  |
| September 1 | Warming | -0.043 | 0.114 | 0.708 | 0.652 | 0.535 | 0.785 | 0.668 | [-0.432, 0.199] | ±0.039 | NA |
|  | Pollution | 0.695 | 0.114 | <0.001*** |  |  |  |  |  |  |  |
|  | Interaction | -0.117 | 0.161 | 0.477 |  |  |  |  |  |  |  |
| September 2 | Warming | -0.013 | 0.041 | 0.747 | 0.277 | 0.218 | 0.387 | 0.328 | [-0.172, 0.055] | ±0.019 | NA |
|  | Pollution | 0.290 | 0.041 | <0.001*** |  |  |  |  |  |  |  |
|  | Interaction | -0.058 | 0.058 | 0.324 |  |  |  |  |  |  |  |

**Table S9:** Diatom concentration GLMs (Gaussian, identity scale).

| **Date** | **Stressor** | **Estimate** | **SE** | **p-value** | **Δadd** | **Δobs** | **Null prediction** | **Observed value** | **Interaction ratio 95% CI** | **±5% band** | **Classification** |
| --- | --- | --- | --- | --- | --- | --- | --- | --- | --- | --- | --- |
| June | Warming | 0.045 | 0.023 | 0.062. | 0.143 | 0.205 | 0.252 | 0.313 | [-0.036, 0.168] | ±0.013 | NA |
|  | Pollution | 0.098 | 0.028 | 0.002** |  |  |  |  |  |  |  |
|  | Interaction | 0.062 | 0.051 | 0.240 |  |  |  |  |  |  |  |

**Table S10**: Green microalgae concentration GLM (June only: Gamma, identity scale).

GAMM model summaries

| **Stressor** | **Estimate** | **SE** | **p-value** | **Δadd** | **Δobs** | **Exponentiated null prediction** | **Exponentiated observed value** | **Exponentiated interaction ratio** | **Interaction ratio 95% CI** | **Classification** |
| --- | --- | --- | --- | --- | --- | --- | --- | --- | --- | --- |
| Warming | -0.887 | 0.214 | <0.001*** | 1.200 | 1.708 | 132.950 | 220.780 | 1.661 | [0.924, 2.984] | Synergism (marginal significance; within ±5% 'additive' boundary) |
| Pollution | 2.087 | 0.210 | <0.001*** |  |  |  |  |  |  |  |
| Interaction | 0.507 | 0.299 | <0.0897. |  |  |  |  |  |  |  |

| **Smooth term** | **EDF** | **df** | **χ²** | **p-value** |
| --- | --- | --- | --- | --- |
| Days since start (Ambient, non-polluted) | 1.000 | 1.000 | 1.437 | 0.231 |
| Days since start (Warm, non-polluted) | 1.000 | 1.000 | 3.913 | 0.048* |
| Days since start (Ambient, polluted) | 1.000 | 1.000 | 0.674 | 0.412 |
| Days since start (Warm, polluted) | 1.802 | 2.236 | 22.352 | <0.001 |

**Table S11:** Barnacle abundance GAMM (log scale).

| **Stressor** | **Estimate** | **SE** | **p-value** | **Δadd** | **Δobs** | **Null prediction** | **Observed value** | **Interaction 95% CI** | **±5% band** | **Classification** |
| --- | --- | --- | --- | --- | --- | --- | --- | --- | --- | --- |
| Warming | -0.182 | 0.134 | 0.177 | -0.231 | -0.034 | 2.822 | 3.019 | [-0.176, 0.569] | ±0.141 | NA |
| Pollution | -0.049 | 0.134 | 0.716 |  |  |  |  |  |  |  |
| Interaction | 0.197 | 0.190 | 0.302 |  |  |  |  |  |  |  |

| **Smooth term** | **EDF** | **df** | **F-statistic** | **p-value** |
| --- | --- | --- | --- | --- |
| Days since start (Ambient, non-polluted) | 1.000 | 1.000 | 34.551 | <0.001*** |
| Days since start (Warm, non-polluted) | 1.000 | 1.000 | 13.377 | <0.001*** |
| Days since start (Ambient, polluted) | 5.186 | 5.747 | 6.434 | <0.001*** |
| Days since start (Warm, polluted) | 3.965 | 4.738 | 2.435 | 0.031* |

**Table S12:** Barnacle size GAMM (Gaussian, identity scale).

| **Stressor** | **Estimate** | **SE** | **p-value** | **Δadd** | **Δobs** | **Null probability** | **Observed probability** | **Odds ratio** | **Interaction ratio 95% CI** | **Classification** |
| --- | --- | --- | --- | --- | --- | --- | --- | --- | --- | --- |
| Warming | -1.640 | 1.225 | 0.181 | 1.225 | 4.962 | 0.294 | 0.950 | 41.984 | [0.259, 6811.937] | NA |
| Pollution | 2.865 | 0.768 | <0.001* |  |  |  |  |  |  |  |
| Interaction | 3.737 | 2.597 | 0.150 |  |  |  |  |  |  |  |

| **Smooth term** | **EDF** | **df** | **χ²** | **p-value** |
| --- | --- | --- | --- | --- |
| Days since start (Ambient, non-polluted) | 0.790 | 6.000 | 3.083 | 0.050* |
| Days since start (Warm, non-polluted) | 0.147 | 6.000 | 0.161 | 0.317 |
| Days since start (Ambient, polluted) | 1.707 | 6.000 | 4.115 | 0.079. |
| Days since start (Warm, polluted) | 3.517 | 6.000 | 9.629 | 0.024* |

**Table S13:** Grazer presence probability GAMM (logit scale).

| **Stressor** | **Estimate** | **SE** | **p-value** | **Δadd** | **Δobs** | **Null proportion** | **Observed proportion** | **Odds ratio** | **Interaction ratio 95% CI** | **Classification** |
| --- | --- | --- | --- | --- | --- | --- | --- | --- | --- | --- |
| Warming | -0.608 | 0.297 | 0.041* | 0.024 | 0.341 | 0.026 | 0.035 | 1.373 | [0.615, 3.064] | NA |
| Pollution | 0.632 | 0.285 | 0.027* |  |  |  |  |  |  |  |
| Interaction | 0.317 | 0.410 | 0.439 |  |  |  |  |  |  |  |

| **Smooth term** | **EDF** | **df** | **χ²** | **p-value** |
| --- | --- | --- | --- | --- |
| Days since start (Ambient, non-polluted) | 4.102 | 4.845 | 37.770 | <0.001*** |
| Days since start (Warm, non-polluted) | 3.644 | 4.376 | 74.220 | <0.001*** |
| Days since start (Ambient, polluted) | 3.869 | 4.622 | 91.930 | <0.001*** |
| Days since start (Warm, polluted) | 2.451 | 3.031 | 37.080 | <0.001*** |

**Table S14:** Macroalgae cover GAMM (logit scale).

| **Stressor** | **Estimate** | **SE** | **p-value** | **Δadd** | **Δobs** | **Null prediction** | **Observed value** | **Interaction 95% CI** | **±5% band** | **Classification** |
| --- | --- | --- | --- | --- | --- | --- | --- | --- | --- | --- |
| Warming | -0.011 | 0.009 | 0.188 | 0.257 | 0.343 | 0.309 | 0.395 | [0.009, 0.162] | ±0.016 | Synergism (within ±5% 'additive' boundary) |
| Pollution | 0.268 | 0.026 | <0.001*** |  |  |  |  |  |  |  |
| Interaction | 0.086 | 0.039 | 0.030* |  |  |  |  |  |  |  |

| **Smooth term** | **EDF** | **df** | **F-statistic** | **p-value** |
| --- | --- | --- | --- | --- |
| Days since start (Ambient, non-polluted) | 1.906 | 2.365 | 4.347 | 0.011* |
| Days since start (Warm, non-polluted) | 1.531 | 1.880 | 3.742 | 0.044* |
| Days since start (Ambient, polluted) | 2.019 | 2.479 | 33.643 | <0.001*** |
| Days since start (Warm, polluted) | 1.000 | 1.000 | 64.495 | <0.001*** |

**Table S15:** Cyanobacteria concentration GAMM (Tweedie, identity scale).

| **Stressor** | **Estimate** | **SE** | **p-value** | **Δadd** | **Δobs** | **Null prediction** | **Observed value** | **Interaction 95% CI** | **±5% band** | **Classification** |
| --- | --- | --- | --- | --- | --- | --- | --- | --- | --- | --- |
| Warming | -0.004 | 0.047 | 0.927 | 0.226 | 0.223 | 0.339 | 0.336 | [-0.133, 0.127] | ±0.017 | NA |
| Pollution | 0.230 | 0.047 | <0.001*** |  |  |  |  |  |  |  |
| Interaction | -0.003 | 0.066 | 0.963 |  |  |  |  |  |  |  |

| **Smooth term** | **EDF** | **df** | **F-statistic** | **p-value** |
| --- | --- | --- | --- | --- |
| Days since start (Ambient, non-polluted) | 1.000 | 1.000 | 1.638 | 0.203 |
| Days since start (Warm, non-polluted) | 1.000 | 1.000 | 0.250 | 0.618 |
| Days since start (Ambient, polluted) | 5.777 | 5.979 | 31.358 | <0.001*** |
| Days since start (Warm, polluted) | 5.818 | 5.986 | 23.559 | <0.001*** |

**Table S16:** Diatom concentration GAMM (Gaussian, identity scale).

Stable isotope analysis (SIA) summaries

| **Treatment** | **Mean δ^15^N** | **SD δ^15^N** | **Mean δ^13^C** | **SD δ^13^C** | **Mean C:N** | **SD C:N** |
| --- | --- | --- | --- | --- | --- | --- |
| Non-polluted + Ambient | 11.705 | 0.288 | -17.235 | 0.221 | 4.107 | 0.083 |
| Non-polluted + Warm | 11.629 | 0.338 | -17.256 | 0.604 | 4.227 | 0.136 |
| Polluted + Ambient | 11.287 | 0.320 | -18.404 | 0.155 | 4.134 | 0.099 |
| Polluted + Warm | 11.371 | 0.275 | -18.343 | 0.166 | 4.210 | 0.079 |
| Alanine (standard) | -1.479 | 0.057 | -27.153 | 0.066 | 2.585 | 0.034 |
| IAEA-CH-6 (standard) | -4.614 | 1.619 | -10.540 | 0.245 | 290.366 | 173.670 |
| Seal collagen (standard) | 16.031 | 0.073 | -12.942 | 0.126 | 2.759 | 0.053 |

**Table S17:** SIA summary statistics.

| **Isotope** | **Stressor** | **Sum of squares** | **Mean of squares** | **F-value** | **p-value** |
| --- | --- | --- | --- | --- | --- |
| δ^15^N | 0.628 | 0.628 | 6.814 | 0.018* | 0.628 |
|  | 0.000 | 0.000 | 0.001 | 0.975 | 0.000 |
|  | 0.035 | 0.035 | 0.379 | 0.546 | 0.035 |
|  | 1.659 | 0.092 | NA | NA | 1.659 |
| δ^13^C | 6.984 | 6.984 | 64.899 | 2.22e-07* | 6.984 |
|  | 0.002 | 0.002 | 0.020 | 0.890 | 0.002 |
|  | 0.009 | 0.009 | 0.086 | 0.772 | 0.009 |
|  | 1.937 | 0.108 | NA | NA | 1.937 |
| C:N ratio | 0.001 | 0.001 | 0.108 | 0.746 | 0.001 |
|  | 0.053 | 0.053 | 5.330 | 0.033* | 0.053 |
|  | 0.003 | 0.003 | 0.267 | 0.612 | 0.003 |
|  | 0.179 | 0.010 | NA | NA | 0.179 |

**Table S18:** SIA ANOVAs.

**Supplementary Figures**

**Methods**

Experimental verifications


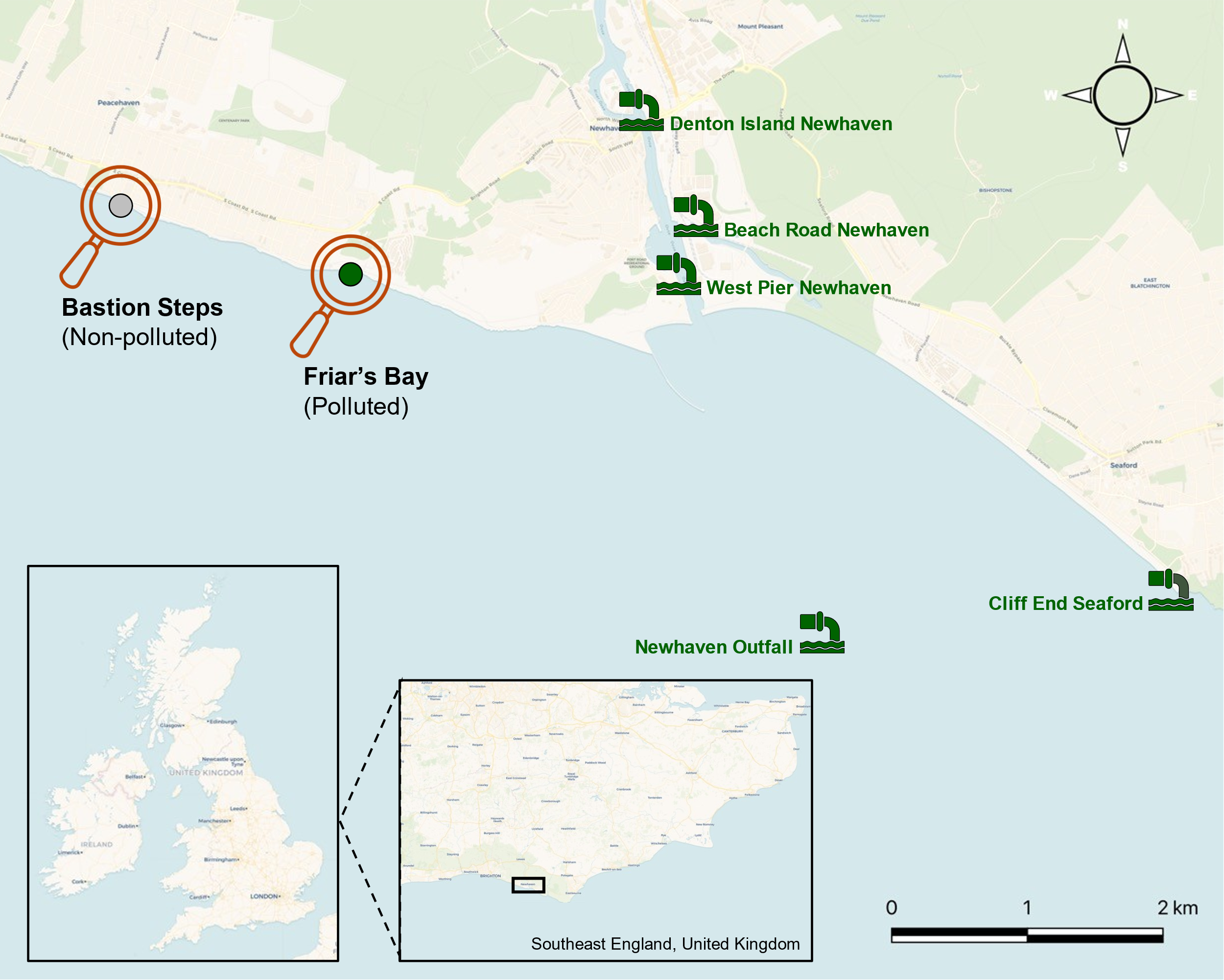


**Figure S1:** Map of Peacehaven and surrounding area, showing installation locations at Bastion Steps (non-polluted) and Friar’s Bay (polluted). Map shows locations of active local sewage outlets, both onshore and offshore.


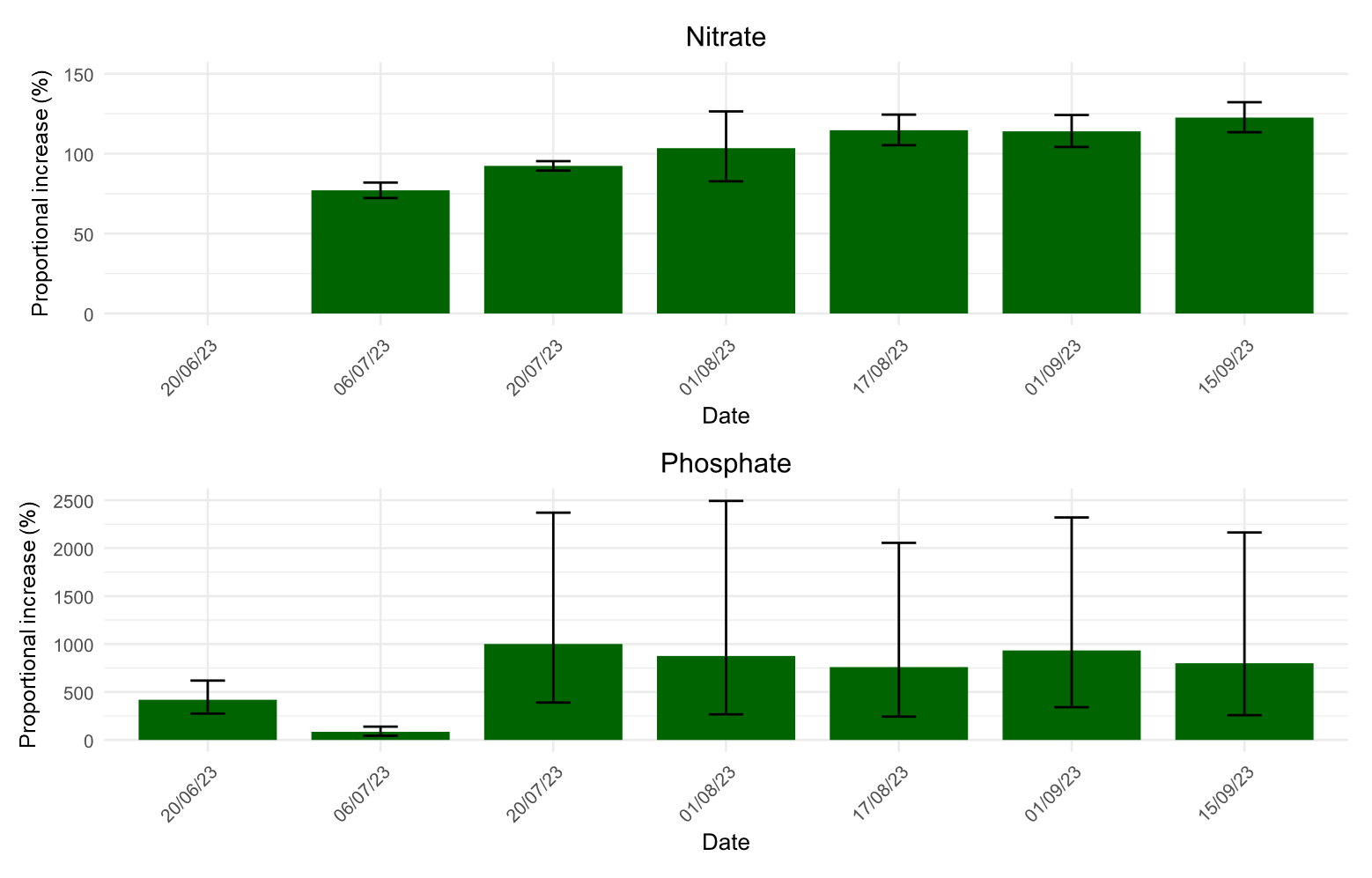


**Figure S2:** Proportional elevation in nitrate and phosphate in the polluted site relative to the non-polluted site throughout the experimental period. For each sampling date and nutrient, five water samples were collected from each site and averaged. Bars show the estimated percentage increase in mean concentration in the polluted site, calculated as 100 × (R - 1), where R is the ratio of polluted to non-polluted mean concentrations on the natural-log scale. Error bars denote 95% confidence intervals derived using the log-ratio delta method from the standard errors of the mean concentrations in each site. Nitrate measurements for 20/06/2023 were omitted due to probe failure.

**Results**

GLM conditional plots


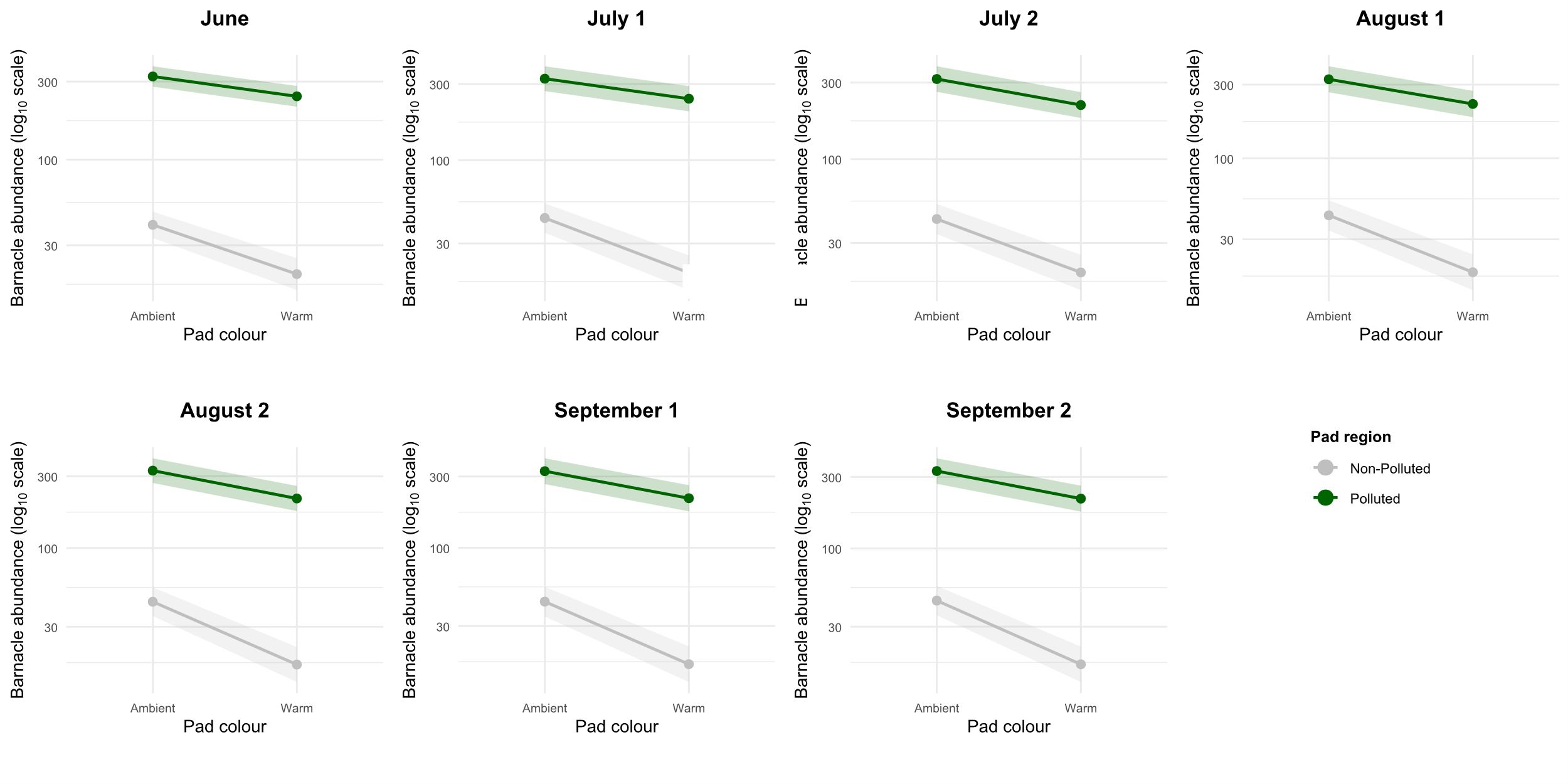


**Figure S3:** Barnacle abundance GLM conditional plots.


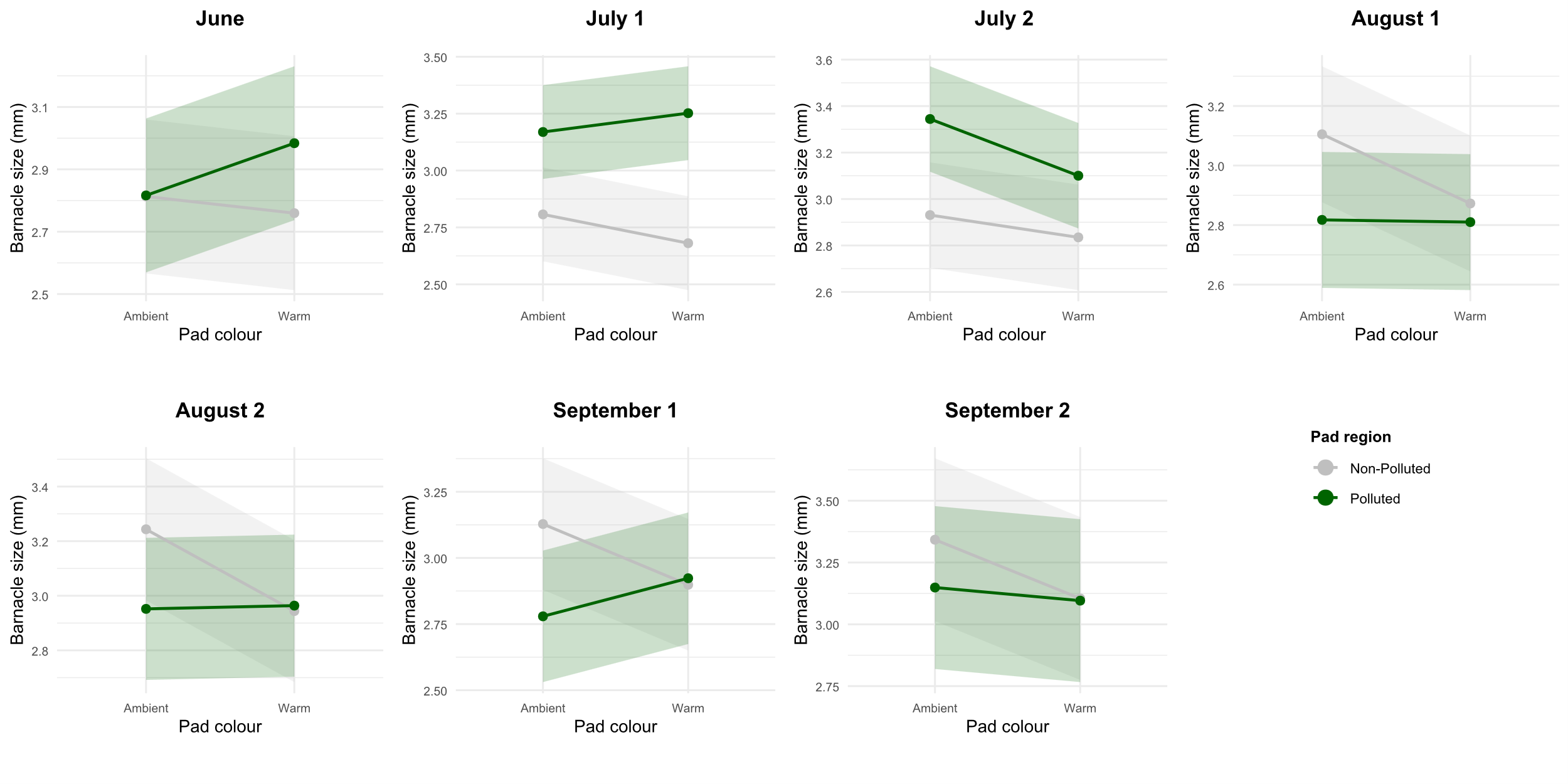


**Figure S4:** Barnacle size GLM conditional plots.

**
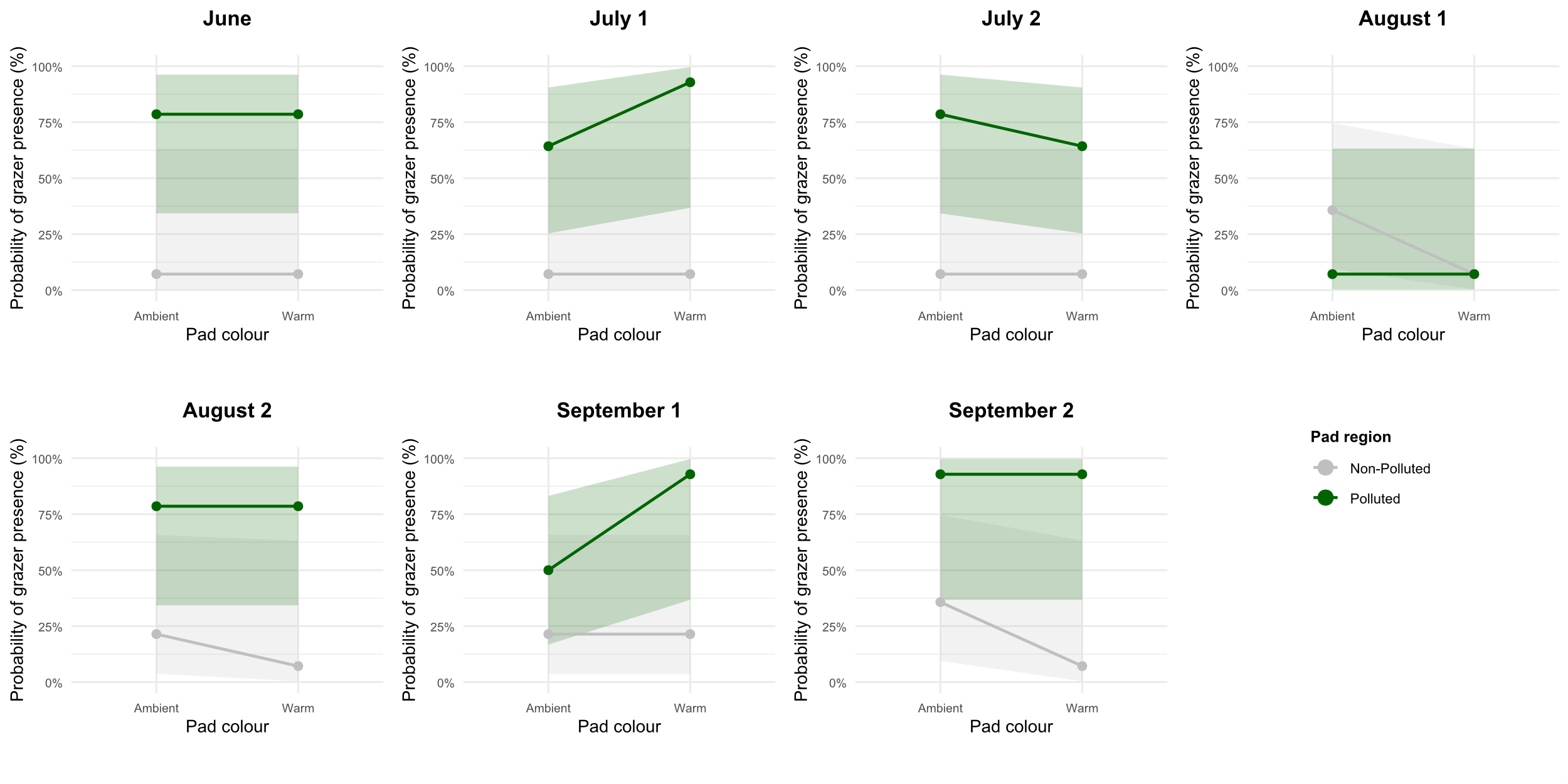
**

**Figure S5:** Grazer presence probability GLM conditional plots.


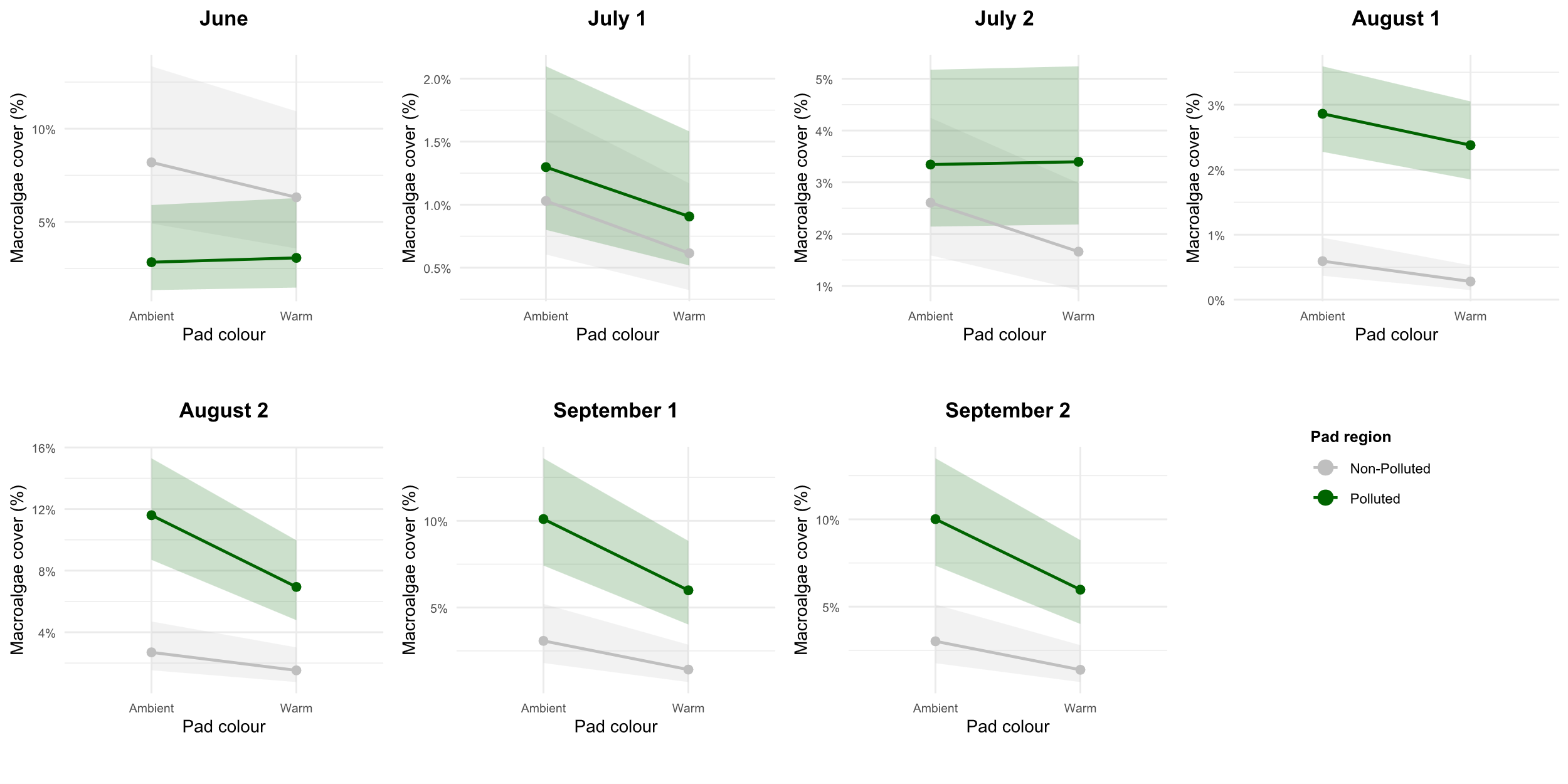


**Figure S6:** Macroalgae cover GLM conditional plots.

**
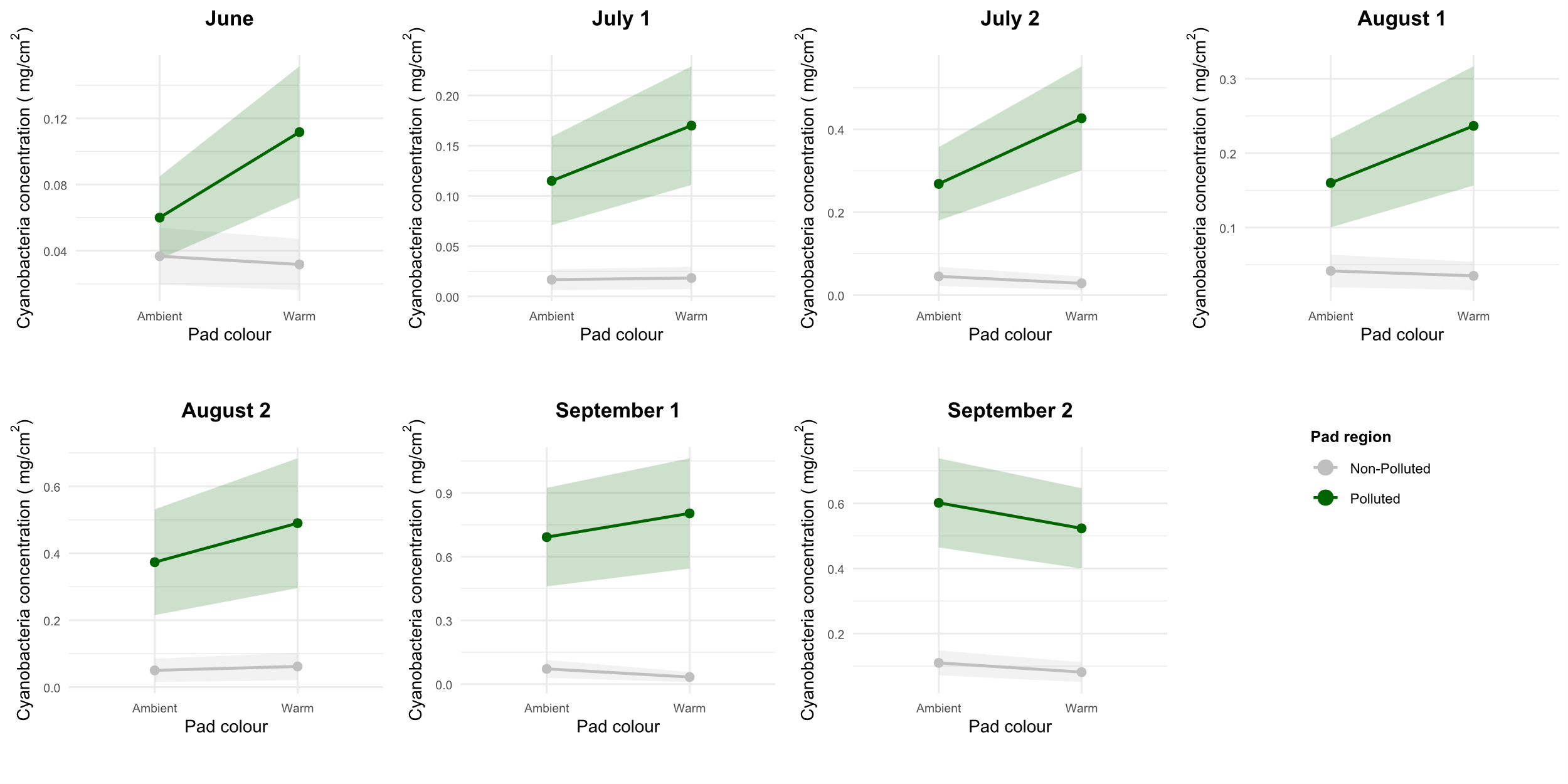
**

**Figure S7:** Cyanobacteria concentration GLM conditional plots.


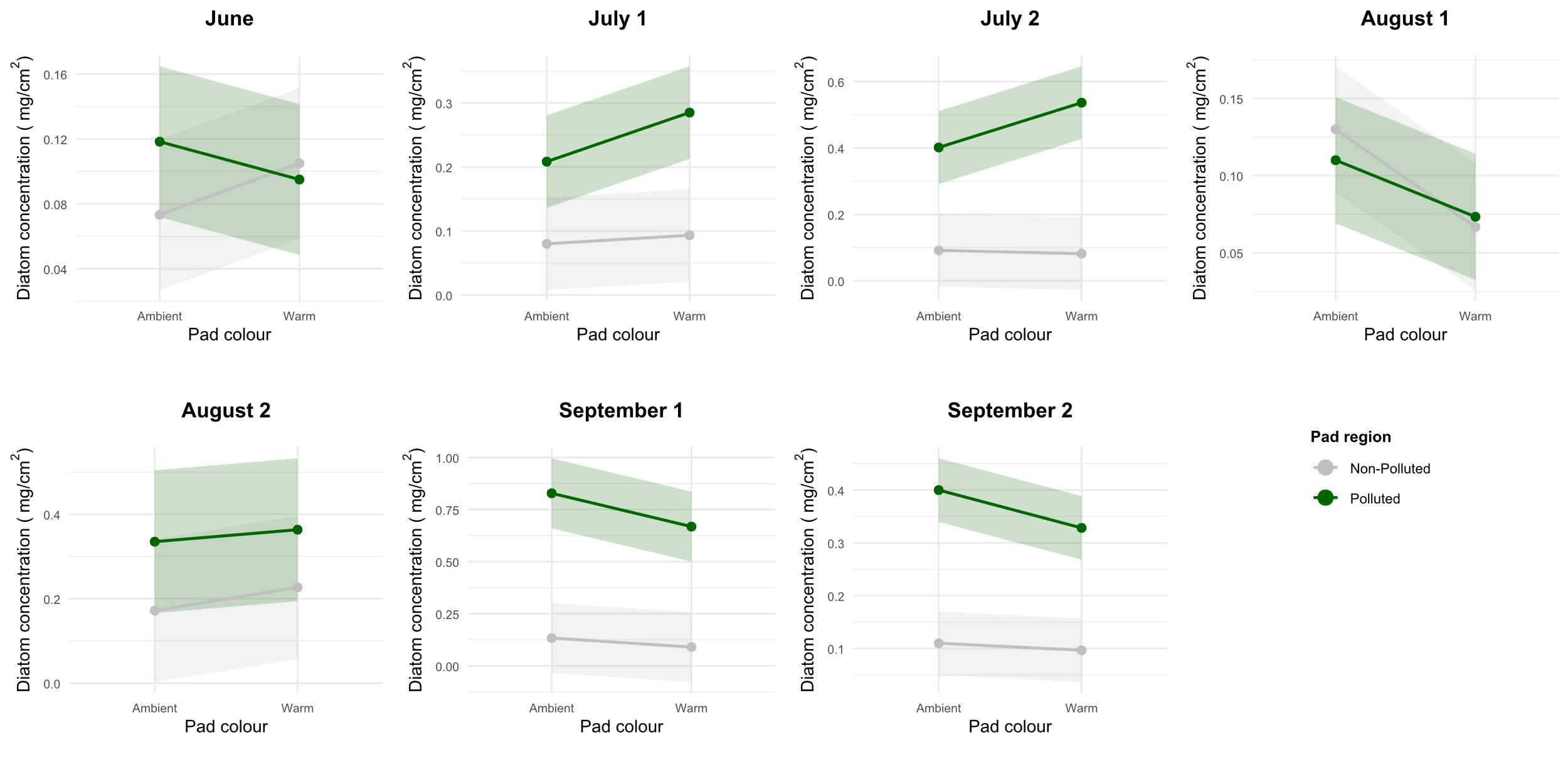


**Figure S8:** Diatom concentration GLM conditional plots.

*
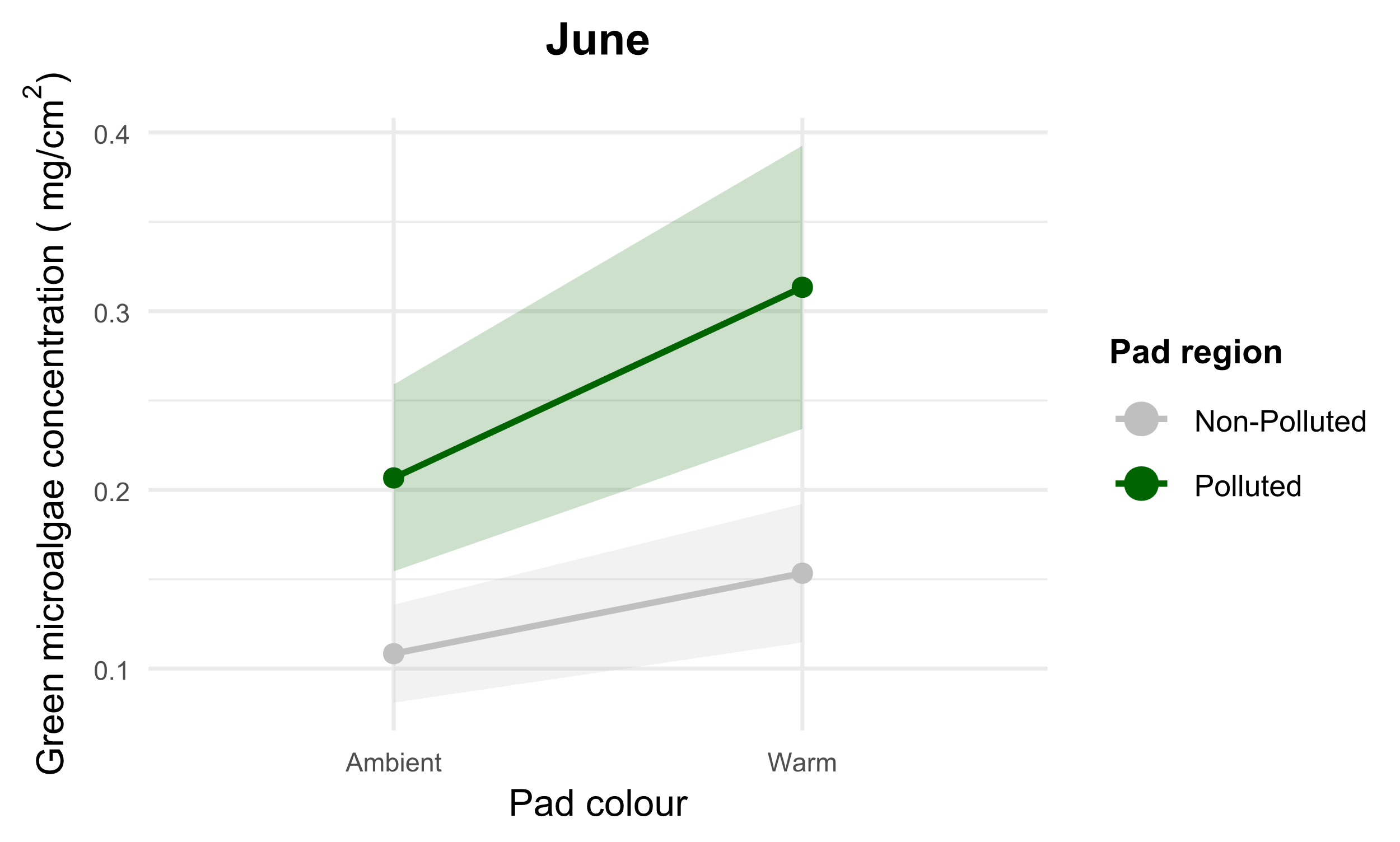
*

**Figure S9:** Green microalgae concentration GLM conditional plot (June only).

GAMM model summaries


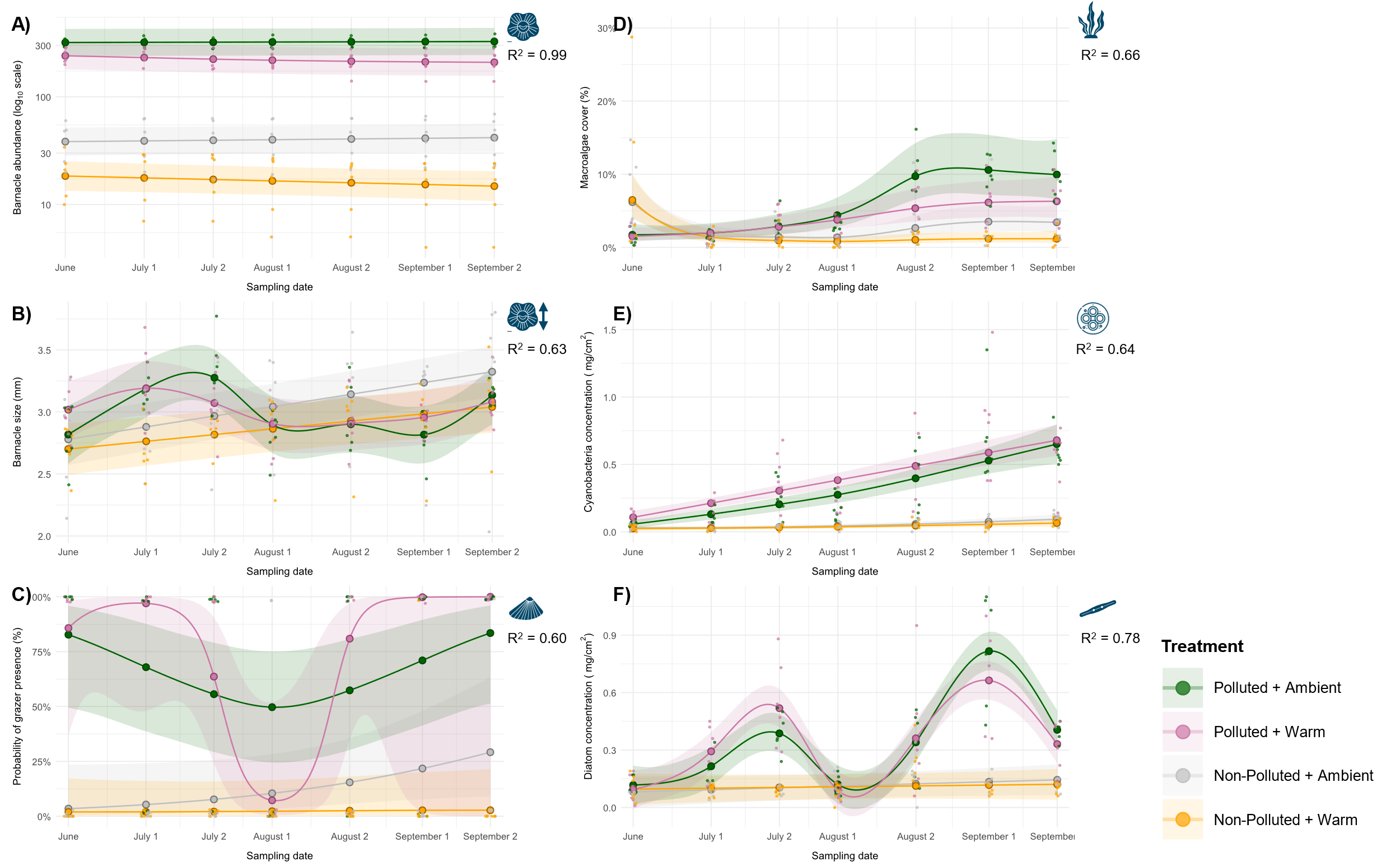


**Figure S10:** Responses over time to warming and nutrient pollution from generalised additive models (GAMMs). Fitted GAMM curves are plotted against days since the start of summer linking large circles representing model estimated means per sampling date, where a smooth term captures potential non-linear temporal trends across stressor combinations throughout the summer season (see ‘GAMM model summaries’ and Table S3 for model details). Shaded ribbons represent 95% CIs, and surrounding individual points are jittered raw data for each replicate to illustrate distributions. The adjusted R^2^ values indicate model fit. A) Barnacle abundance; B) Barnacle size; C) Grazer presence; D) Macroalgae cover; E) Cyanobacteria concentration; F) Diatom concentration.


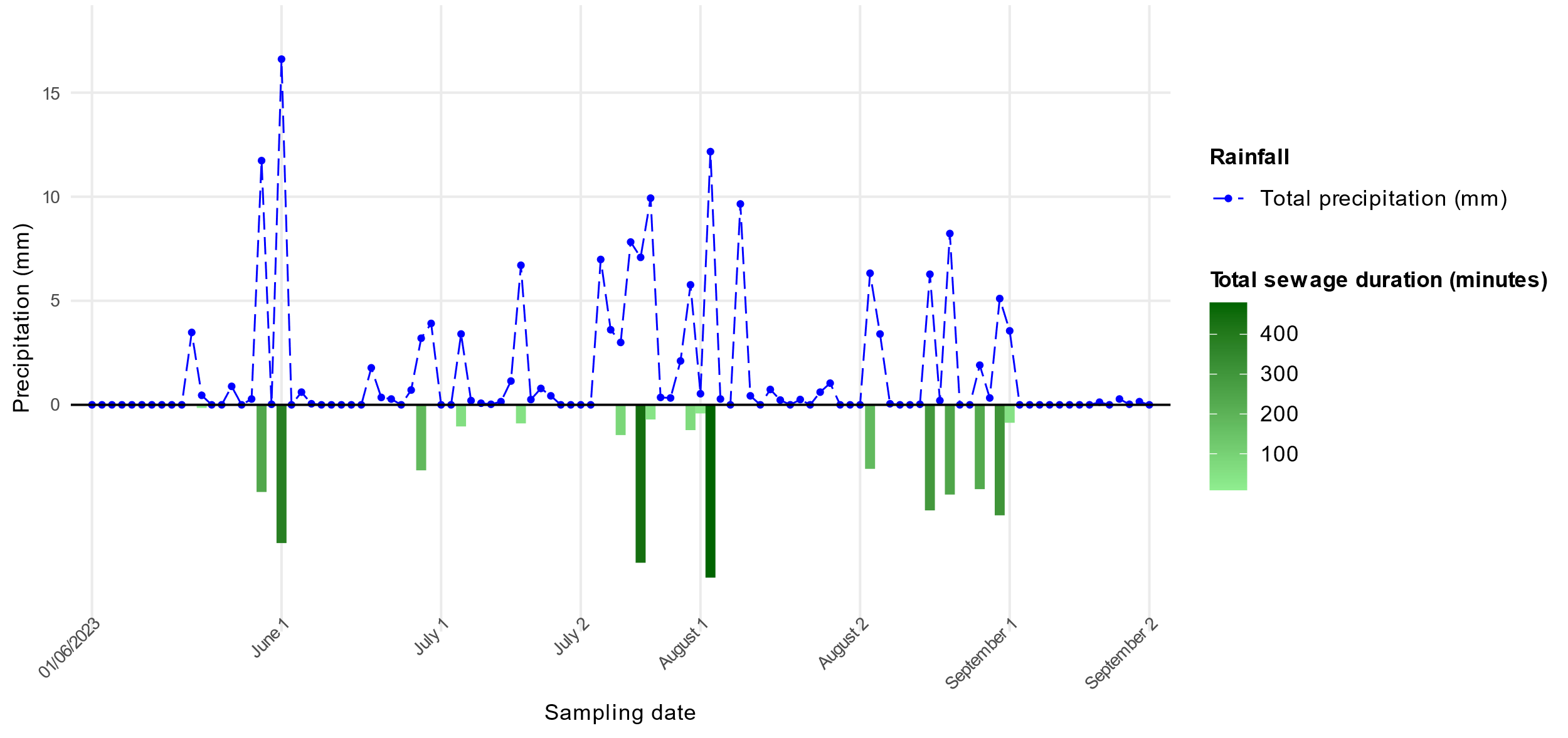


**Figure S11:** Comparison of precipitation and sewage release events. Precipitation data for Peacehaven sourced from Visual Crossing database, showing daily precipitation (mm). Heat mapped bars indicate the total daily duration of sewage release events at Newhaven Outfall (see Figure S1), with duration (minutes) indicated by colour and length of bar. Figure shows from June 1^st^ 2023, prior to the June 1 sampling date (June 20th) for additional context.

**Supplementary References**

- Denny, M. W. & Harley, C. D. G. Hot limpets: predicting body temperature in a conductance-mediated thermal system. *J. Exp. Biol.* 209, 2409–2419 (2006).
- Kosmidis, I. & Firth, D. Jeffreys-prior penalty, finiteness and shrinkage in binomial-response generalized linear models. *Biometrika* 108, 71–82 (2021).
- Kordas, R. L., Dudgeon, S., Storey, S. & Harley, C. D. G. Intertidal community responses to field-based experimental warming. *Oikos* 124, 888–898 (2015).
- Kordas, R. L. & Harley, C. D. G. Demographic responses of coexisting species to in situ warming. *Mar. Ecol. Prog. Ser.* 546, 147–161 (2016).
- Kordas, R. L., Donohue, I. & Harley, C. D. G. Herbivory enables marine communities to resist warming. *Sci. Adv.* 3, e1701349 (2017).
- LaScala-Gruenewald, D. E. & Denny, M. W. Long-term mechanistic hindcasts predict the structure of experimentally-warmed intertidal communities. *Oikos* 129, 1645–1656 (2020).
- López-Maury, L., Marguerat, S. & Bähler, J. Tuning gene expression to changing environments: from rapid responses to evolutionary adaptation. *Nat. Rev. Genet.* 9, 583–593 (2008).
- Sleight, V. A., Peck, L. S., Dyrynda, E. A., Smith, V. J. & Clark, M. S. Cellular stress responses to chronic heat shock and shell damage in temperate *Mya truncata*. *Cell Stress Chaperones* 23, 1003–1017 (2018).
- Spake, R. et al. Understanding ‘it depends’ in ecology: a guide to hypothesising, visualising and interpreting statistical interactions. *Biol. Rev.* 98, 983–1002 (2023).
- Wood, S. N. Generalized additive models. *Annu. Rev. Stat. Appl.* 12, 497–526 (2025).
